# Smoking prevalence following the announcement of tobacco tax increases in England between 2007 and 2019: an interrupted time–series analysis

**DOI:** 10.1111/add.15898

**Published:** 2022-05-01

**Authors:** Emma Beard, Jamie Brown, Lion Shahab

**Affiliations:** ^1^ Research Department of Behavioural Science and Health University College London London UK; ^2^ SPECTRUM Consortium London UK

**Keywords:** Cigarette consumption, cost, England, quit attempts, quit success, smoking, tax

## Abstract

**Aims:**

This study aimed to evaluate the impact of announcement of tax increases on factory‐made (FM) and roll‐your own (RYO) cigarettes in England.

**Design, Setting and Participants:**

Autoregressive integrated moving average with exogeneous input (ARIMAX) time–series modelling in England, UK. Data were aggregated monthly on 274 890 participants between 2007 and 2019 taking part in the Smoking Toolkit Study (STS).

**Measurements:**

The association of sustained step level changes for tax rises for FM cigarettes and temporary pulse effects for tax rises for RYO cigarettes with smoking, quit attempt and quit success prevalence as well as per‐capita self‐reported cigarette consumption and cost per cigarette was assessed.

**Findings:**

A 10% rise in tax on RYO cigarettes was associated with a temporary 21.1% decline [95% confidence interval (CI) = –30.4 to −10.7] in smoking prevalence, and 20.7% decline (95% CI = –32.4 to −7.0) in per‐capita self‐reported cigarette consumption; while a 3% rise of tax on RYO cigarettes was associated with a temporary 20.7% decline (95% CI = –33.3 to −5.8) in the amount paid per RYO cigarette. For tax increases on FM cigarettes, a 5% above inflation tax rise was associated with a step‐level increase of 33.1% (95% CI = 18.4–49.5) in quit success rates. However, some of the findings were sensitive to model specification and temporally specific.

**Conclusion:**

The announcements of tax increases for cigarettes in England between 2010 and 2019 were inconsistently associated with temporary reductions in smoking prevalence, per‐capita self‐reported cigarette consumption and improved quit success. Paradoxically, reductions in the cost for roll‐your‐own cigarettes were also found. The results were not robust in all sensitivity analyses.

## INTRODUCTION

England has a strong tobacco control climate, as evidenced by the introduction of the first national tobacco control strategy ‘Smoking Kills’ in 1998. This strategy resulted in a range of policies aimed at increasing the retail price of cigarettes. These included a rise in taxation [1, 2] and the Minimum Excise Tax (MET) which ensures a minimum price for tobacco and minimum pack size (i.e. of 20 cigarettes) and therefore high purchase price [3, 4]. Studies throughout several countries have shown that tax increases are cost‐effective interventions for reducing smoking prevalence by promoting quitting and reducing uptake, with those in lower socio‐economic groups being most responsive [5, 6].

Data are available from the Smoking Toolkit Study (STS), a population survey of adults aged 16+ in England, to evaluate the impact of tax increases in England [7]. For the first three years after the STS was established in November 2006 taxes rose in line with inflation, as measured by the Retail Prices Index (RPI). In 2009, tobacco duties were increased to ensure that the overall level of taxation remained broadly unchanged following a temporary reduction in VAT, while in 2010 taxation was 1% above inflation. A duty escalator followed this in 2011 at 2% above inflation, with an additional 10% increase on roll‐your own (RYO) tobacco. Duty was increased by 5% above inflation in the 2012 budget, and in the 2013 budget the tax rise reverted to 2% above inflation. In 2016, tax on RYO tobacco increased by 5% above inflation and in 2018 by 3% above inflation (see Table 1) [8].

**TABLE 1:** Time‐line of tax increases on tobacco in England from January 2007 until December 2019

Date	FM cigarettes	RYO
	Tax increase	Tax increase
January 2007–February 2007	In line with inflation	
March 2007–February 2008	In line with inflation	
March 2008–February 2009	In line with inflation	
March 2009–February 2010	2% increase (overall level of taxation remained broadly unchanged following the temporary reduction in VAT)	
March 2010–February 2011	1% above inflation	
March 2011–February 2012	2% above inflation	10% increase
March 2012–February 2013	5% above inflation	
March 2013–February 2014	2% above inflation	
March 2014–February 2015	2% above inflation	
March 2015–June 2015	2% above inflation	
July 2015–February 2016	2% above inflation	
March 2016–February 2017	2% above inflation	5% above inflation
March 2017–October 2017	2% above inflation	
November 2017–October 2018	2% above inflation	3% above inflation
November 2018–December 2019	2% above inflation	3% above inflation

RYO = roll‐your‐own; FM = factory‐made; VAT = value‐added tax. Budget 2006 http://webarchive.nationalarchives.gov.uk/20130129110402/http://www.hmtreasury.gov.uk/d/bud06_completereport_2320.pdf.

Budget 2007 http://webarchive.nationalarchives.gov.uk/20130129110402/http://www.hmtreasury.gov.uk/d/bud07_completereport_1757.pdf.

Budget 2008 https://www.gov.uk/government/uploads/system/uploads/attachment_data/file/250345/0388.pdf.

Budget 2009 http://webarchive.nationalarchives.gov.uk/20130129110402/http://www.hmtreasury.gov.uk/d/bud09_completereport_2520.pdf.

Budget 2010 http://webarchive.nationalarchives.gov.uk/20130129110402/http://www.hmtreasury.gov.uk/d/budget2010_complete.pdf.

Budget 2011 http://webarchive.nationalarchives.gov.uk/20130129110402/http://www.hmtreasury.gov.uk/2011budget.htm.

Budget 2012 http://webarchive.nationalarchives.gov.uk/20130129110402/http:/www.hmtreasury.gov.uk/budget2012.htm.

Budget 2013 https://www.gov.uk/government/publications/budget‐2013‐documents.

Budget 2014 https://www.gov.uk/government/topical‐events/budget‐2014.

Budget 2015 https://www.gov.uk/government/publications/budget‐2015‐documents2.

Summer Budget 2015 https://www.gov.uk/government/topical‐events/budget‐july‐2015.

Budget 2016 https://www.gov.uk/government/topical‐events/budget‐2016.

Budget 2017 (March) https://www.gov.uk/government/publications/spring‐budget‐2017‐documents.

Budget 2017 (Nov) https://www.gov.uk/government/topical‐events/autumn‐budget‐2017.

Budget 2018 https://www.gov.uk/government/news/budget‐2019‐date‐announced.

Recently, an empirical study assessed the immediate and longer‐term effects of tax increases on the prevalence of smoking in Australia [9], finding that large increases (25%) were effective in reducing smoking prevalence. The authors used an interrupted time–series design to account for confounding by other tobacco control policies and secular trends. Similarly, time–series analyses of US data found that an increase in the price of cigarettes to more than $4 per 20‐cigarette pack was associated with a significant decrease in smoking among younger people [10]. Decreases were also seen in asthma hospitalizations, acute myocardial infarction hospitalization and sudden cardiac death rates. In other studies, significant increases in tobacco taxes have been shown to encourage current tobacco users to stop using and reduce consumption among those who continue to use, with the greatest impact on the young and disadvantaged groups [6].

We aimed to apply this methodology in England and assess the possible impact of announcement of tax rises on:

- current smoking prevalence
- quit attempts among past year smokers
- the success of those quit attempts
- per‐capita self‐reported cigarette consumption per day among the population
- cost per cigarette among exclusive RYO cigarette smokers
- cost per cigarette among exclusive factory‐made (FM) smokers.

Results are presented overall and stratified by socio‐economic status (SES), with the prediction of greater effects among lower SES. Sensitivity analyses were also run to determine the impact of these tax increases on prevalence of smoking of predominant FM cigarette and prevalence of smoking of predominant RYO cigarettes. An additional sensitivity analysis determined the impact of tax increases on the average consumption of exclusively smoked FM cigarettes and exclusively smoked RYO cigarettes.

## METHODS

Full details of the methodology and analysis can be found in the Supporting information. The Strengthening the Reporting of Observational Studies in Epidemiology (STROBE) guidelines were followed throughout [11].

### Study design

Data come from the Smoking Toolkit Study (STS), a monthly survey of a representative sample of the population in England aged 16+. Participants from the STS appear to be representative of the population in England, having similar socio‐demographic composition as other large national surveys, such as the Health Survey for England and retail sales data [7, 12]. Data were used between January 2007 and December 2019. Although the STS was established in 2006 and is ongoing, data on several of the covariates, including mass media expenditure, were only available during this period.

### Data on outcome variables

#### Prevalence of smoking, quit attempts and the success of quit attempts

Smoking prevalence, quit attempt prevalence and the prevalence of the success of those quit attempts were derived using the following questions:

- ‘Which of the following best applies to you? (a) I smoke cigarettes (including RYO) every day; (b) I smoke cigarettes (including RYO), but not every day; (c) I do not smoke cigarettes at all, but I do smoke tobacco of some kind (e.g. pipe or cigar); (d) I have stopped smoking completely in the last year; (e) I stopped smoking completely more than a year ago; (f) I have never been a smoker (i.e. smoked for a year or more)’.
- [Past‐year smokers only] ‘How many serious attempts to stop smoking have you made in the last 12 months? By serious attempt I mean you decided that you would try to make sure you never smoked again. Please include any attempt that you are currently making and please include any successful attempt made within the last year’.
- [Past‐year smoker who have made a quit attempt only] ‘How long did your most recent serious quit attempt last before you went back to smoking?’

The prevalence of current cigarette smoking was calculated as the proportion of respondents who report (ia) or (ib). The prevalence of quit attempts in each month was calculated as the number of respondents who reported having made one or more quit attempts in the past 12 months divided by the number of past year smokers in response to (ii).

The quit success rate in each month was calculated as the number of respondents reporting that they were still not smoking divided by the number reporting having made a quit attempt in response to (iii).

Two sensitivity analyses used the (a) smoking prevalence of predominant FM cigarette and (b) smoking prevalence of predominant RYO cigarette instead of overall cigarette smoking prevalence. Cigarette smokers were asked how many RYO cigarettes they smoked per day. The prevalence of predominant roll‐your‐own smoking in each month was calculated as the proportion of participants who report that ≥ 50% of the cigarettes they smoked are roll‐your‐own cigarettes [13]. The prevalence of predominant FM cigarette smoking in each month was calculated as the proportion of participants who report that < 50% of the cigarettes they smoked were RYO cigarettes.

#### Per‐capita self‐reported cigarette consumption

Smokers were asked on average how many cigarettes they smoked per day. The per‐capita consumption per day was then calculated as the summation of cigarettes smoked per day (which was set to zero for all non‐current smokers, i.e. non‐smokers and ex‐smokers) divided by the entire population. Consumption within the population rather than among smokers was used to reflect quitting, i.e. a reduction may reflect an increase in recent ex‐smokers without a change in consumption by continuing smokers.

The mean per‐capita consumption was calculated separately per day for (i) FM cigarettes and (ii) RYO cigarettes. FM conusmption was calculated as the summation of cigarettes smoked per day among those exclusively smoking FM cigarettes divided by cigarette consumption for the entire population, which was set to 0 for all non‐current exclusive FM cigarette smokers; RYO consumption was calculated as the summation of cigarettes smoked per day among those exclusively smoking RYO cigarettes divided by cigarette consumption for the entire population, which was set to 0 for all non‐current exclusive RYO cigarette smokers.

#### Cost per cigarette (£)

To assess self‐reported cost per cigarette, current smokers were asked: ‘On average about how much per week do you think you spend on cigarettes or tobacco?’ and the number of cigarettes they smoked per week was calculated (including hand‐rolled). Smokers’ average expenditure of smoking (in £/week) was derived from the following liberal assumptions for upper and lower estimates of plausible levels of consumption and expenditure per week [14]: (1) consumption of a maximum of 560 cigarettes per week; (2) spending does not exceed £280 per week; and (3) single cigarette expenditure between £0.05 and £1. The cost of smoking was adjusted for inflation using Consumer Prices Index data of all items from the Office for National Statistics, with January 2007 as the baseline/reference. The total cost per week was divided by the total number of cigarettes smoked per week to give an estimate of cost per cigarette. Cost per cigarette (£) was derived separately for those exclusively smoking FM cigarettes and those exclusively smoking RYO cigarettes. Cost per cigarette was stratified in this way as any associations between tax increases and cigarette cost may be diluted by smokers switching between FM and RYO cigarettes, depending on the nature of the tax increase.

### Data on covariates

Covariates were chosen based on there being a plausible relationship between the variables and the outcomes of interest. The covariates included several tobacco control policies combined into a composite score (coded 1 during the month they were implemented and 0 in other months). These were the introduction of a smoking ban in July 2007, change in the minimum age of sale of cigarettes in October 2007, pictorial health warnings on product packaging introduced in October 2008, partial (i.e. supermarket) tobacco point‐of‐sale display ban introduced in England in April 2012, the full point‐of‐sale ban in April 2015 and the tobacco products directive/plain packaging in May 2016.

We also included monthly tobacco mass media expenditure (in £million) which was obtained from Public Health England. Total spending on campaigns was calculated for each month and included spending on ‘Smokefree’ campaigns, Stoptober campaigns and Health Harms campaigns. Spending included TV, radio, print, cinema and on‐line advertisements. In England, tobacco control mass media campaigns have been run as part of a national tobacco control programme. Spending was almost completely suspended in 2010 and then re‐introduced in 2011 at a much lower level.

### Data on explanatory variables

We modelled the date of the announcement of the tax increases rather than the implementation. Tobacco industry tactics are implemented following the announcement: increasing prices on top of tax increases, so that both the price and tax increase are passed on to consumers (known as overshifting), absorbing the tax increase so it is not passed on to consumers (undershifting) or passing the tax increase on to consumers in full (fully shifting) [15]. Thus, modelling the annoucmenet means that the impact of these immediate tactics on smoking behaviour would be detected. Moreover, the announcement of the tax rise is likely to be the moment where consumers become aware of the cost of cigarettes going up and is therefore a useful benchmark.

For the overall primary analysis, we modelled two step level changes (sustained effects) for the tax increases on FM cigarettes: (1) the shift to above inflation tax increases on FM cigarettes from March 2010 and (2) the increase of 5% above inflation in March 2012 on FM cigarettes. For both, the first segment of data covers a period of only inflation adjustment with no real increases in tobacco tax (January 2007 to February 2010), this period was thus coded 0. The first step level change was for the period of above inflation tax increases between March 2010 until December 2019. This period was coded as 1. The second step level change reflected the larger tax increase of 5% in March 2012 and was coded 0 before February 2012 and 1 thereafter. We also included four pulse effects for the tax increases on RYO cigarettes (coded 1 during the month they were first implemented and 0 in other months): in March 2011, March 2016, November 2017 and November 2018. The decision was made a priori to measure these as pulse effects as they were not followed by further immediate tax increases. Temporary pulse effects may more reflect the announcement forewarning smokers about tax changes, while step level changes may reflect more of the impact of tax increases themselves or tobacco industry tactics.

As a sensitivity analysis, we also modelled the 5% increase in tax on FM cigarettes as a pulse effect which was coded 1 for March 2012 and 0 for all other months. We also included a step level effect for the four time points in which tax increases occurred for RYO tobacco (coded 0 before the tax change and 1 after, see data file). Sensitivity analyses stratified results by use of FM cigarettes and RYO cigarettes and by SES.

Occupation social‐grade (ABC1 versus C2, D, E) was used to stratify the sample by SES.

## ANALYSIS

### Amendments

In the original analysis plan we had included ever smoking (as an indicator of uptake) as an outcome variable. However, effect size estimates from the models including ever smoking appeared implausibly large. These data are provided on the Open Science Framework, together with the results of the pre‐planned analysis for ever smoking (https://osf.io/kz3bc/).

During the same internal review, it was also decided to add new outcome variables in the primary and sensitivity analyses: (i) average population cigarette consumption (primary analysis), (ii) average population FM and RYO cigarette consumption per day (sensitivity analysis) and (iii) average cost per FM and RYO cigarette (primary analysis). These variables may be more sensitive to FM/RYO‐specific tax increases and have been used widely in previous literature [6, 14, 16, 17].

Following concerns that the size of the significant pulse and step level effects may have been sensitive to randomness in the monthly data, it was also decided to run additional unplanned sensitivity analyses using smoothed data at the quarterly rather than monthly level.

### Sample size

Power simulations assuming 156 months of data collection, a step level change occurring in month 39 (March 2010) and month 63 (March 2012) and baseline smoking prevalence of 24.3% and autoregressive autocorrelation of lag‐1 (value 0.5) indicated that we had 80% power to detect a step level change of 1 percentage point.

### Primary analysis

All data were analysed in R studio [18]. The data frame and analysis plan were pre‐registered on the Open Science Framework (https://osf.io/kz3bc/). Alpha was set to 0.05; 95% confidence intervals (CIs) are reported.

We used an interrupted time–series analysis to account for autocorrelation among monthly observations by fitting autoregressive integrated moving average with exogeneous input (ARIMAX) models [19, 20]. ARIMAX is an extension of autoregressive integrated moving average analysis (ARIMA), which produces forecasts based upon prior values in the time series analysis (AR terms) and the errors made by previous predictions (MA terms). Both adjusted and unadjusted models are reported in this paper. To identify the most appropriate transfer function for the continuous explanatory variables, we checked the sample cross‐correlation function and compared models with varying lags using the Akaike information criterion.

For the primary analysis, we modelled step level changes as a result of the rise in tax on FM cigarettes and pulse effects as a result of the rise in tax on RYO. We pre‐specified modelling the tax increases in this way as: (1) tax increases for RYO were infrequent and therefore we hypothesized temporary rather than longer‐term changes, and (2) tax increases for FM were frequent and consistent over time and therefore were better modelled as step changes. In the analysis these models were also the most stable and provided an adequate fit.

Bayes factors were calculated for non‐significant findings for the primary analysis in R using code described by Dienes [21, 22]. This helps to determine if there is evidence for the null hypothesis of no difference or the data are insensitive to detect an effect.

Ethical approval for the Smoking Toolkit Study was granted by the UCL ethics committee (ID 0498/001). The data are not collected by UCL and are anonymized when received by UCL. Participants provided full informed consent.

### Availability of data and materials

The data frame was pre‐registered on the Open Science Framework (https://osf.io/kz3bc/).

## RESULTS

Individual‐level data were aggregated monthly between January 2007 and December 2019 on 274 890 participants, of whom 19.6% [95% confidence interval (CI) = 19.5–19.8] were current smokers and 21.6% (95% CI = 21.4–21.7) were past year smokers. Of the past year smokers, 35.5% (95% CI = 35.1–35.9) had made a quit attempt in the past year, 15.8% (95% CI = 15.2–16.3) of which were successful.

**FIGURE 1:**
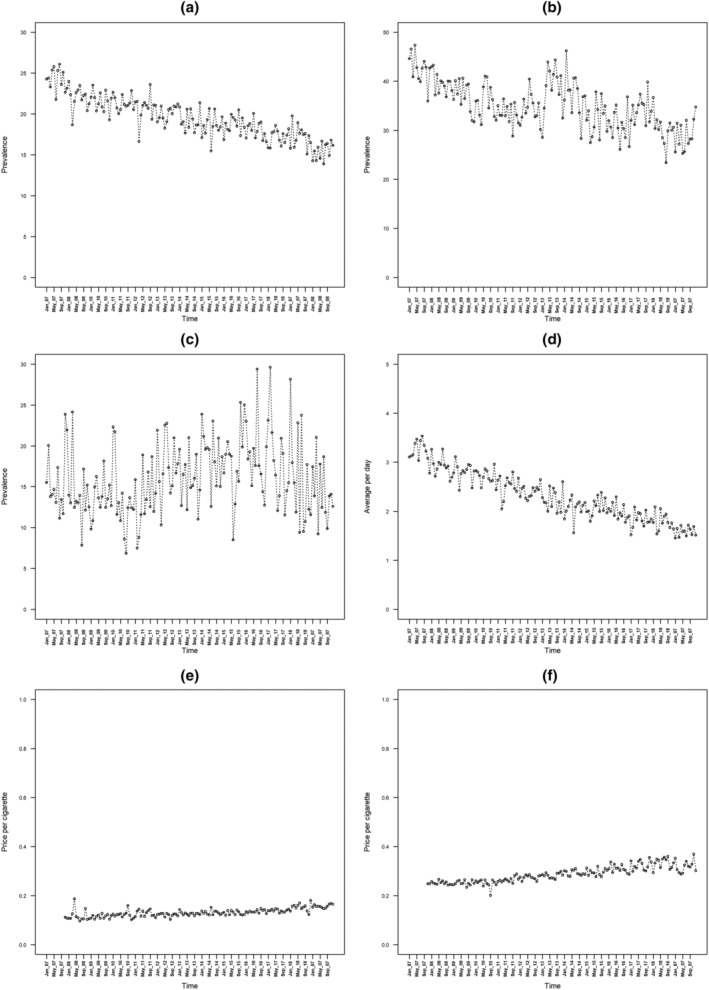
Prevalence of (a) smoking, (b) quit attempts among past‐year smokers and (c) quit success among past year smokers having made a quit attempt, (d) per‐capita self‐reported cigarette consumption per day, (e) cost per cigarette among exclusive roll‐your‐own (RYO) smokers and (f) cost per cigarette among exclusive factory‐made (FM) cigarettes

During the study period, smoking prevalence declined from 24.3% (95% CI = 22.2–26.3) in January 2007 to 16.1% (95% CI = 14.4–17.8) in December 2019. A decline in the number of smokers making a quit attempt [from 44.6% (95% CI = 39.8–49.9%) to 34.8% (95% CI = 31.1–38.5)] and in the quit success rate [from 15.5% (95% CI = 12.0–19.0) to 12.6% (95% CI = 10.0–15.2)] was also seen during the same period. These declines are probably partially attributable to the high levels of quitting activity at the start of the series due to the impending smoking ban in 2007. Daily self‐reported cigarette consumption in the whole population declined over the study period from 3.1 (95% CI = 2.8–3.4) to 1.5 (95% CI 1.3–1.8). Cost per cigarette increased slightly from £0.25 to £0.30 for FM and from £0.11 to £0.16 for RYO during the course of the study. Figure 1 shows the prevalence of smoking, quit attempts, the success of quit attempts, per‐capita self‐report cigarette consumption and the cost per FM/RYO cigarette during the course of the study.

### Effect of RYO‐targeted tax increases (pulse effects)

#### Primary analysis

In adjusted models, there was a significant association between a temporary pulse in the rise in tax by 10% on RYO cigarettes in March 2011 and smoking prevalence (see Table 2) and per‐capita self‐reported cigarette consumption per day (see Table 3). It was estimated that the rise in tax was associated with a 21.1% decline in smoking prevalence and 20.7% decline in per‐capita self‐reported cigarette consumption per day. Fitted values from the model indicated that this equated to reducing smoking prevalence from 21.3 to 16.8% from before to after the tax rise (from February 2011 to March 2011) and reducing self‐reported cigarette consumption per day from 2.7 to 2.1.

**TABLE 2:** Association between tax increases and overall smoking prevalence, quit attempt prevalence and quit success prevalence (adjusted for all tax policies, mass media and tobacco control policies)

	Model 1: smoking prevalence				Model 2: quit attempt prevalence				Model 3: quit success prevalence			
	B	95% CI		*P*	B	95% CI		*P*	B	95% CI		*P*
		Lower	Upper			Lower	Upper			Lower	Upper	
Step level change 1 for FM (March 2010–December 2012)	−4.1	−11.5	4.0	0.311	0.2	−12.7	15.0	0.979	−9.7	−21.2	3.6	0.144
Step level change 2 for FM (March 2012–December 2019)	−2.9	−10.4	5.3	0.482	9.9	−4.3	26.1	0.180	33.1	18.4	49.5	< 0.001
Pulse effect for RYO (March 2011)	−21.1	−30.4	−10.7	< 0.001	−3.2	−19.4	16.1	0.720	−31.5	−58.3	12.3	0.133
Pulse effect for RYO (March 2016)	−3.8	−8.2	17.8	0.539	−11.0	−25.9	6.8	0.211	7.8	−3.2	20.1	0.174
Pulse effect for RYO (November 2017)	2.1	−9.9	15.7	0.745	−6.6	−22.4	12.4	0.473	−23.7	−53.4	25.0	0.282
Pulse effect for RYO (November 2018)	5.9	−6.7	20.0	0.377	3.9	−13.7	25.0	0.687	−17.1	−36.7	8.4	0.171
Model specification	ARIMAX (0,1,1)				ARIMAX (0,1,1)				ARIMAX (0,1,1)			
AR												
MA	−0.799	−0.885	−0.712	< 0.001	−0.721	−0.846	−0.597	< 0.001	−1.000	−1.038	−0.962	< 0.001
SAR												
SMA												

AR = autoregressive; MA = moving average; SAR = seasonal autoregressive; SMA = seasonal moving average; ARIMAX = autoregressive integrated moving average with explanatory variable (ARIMAX); RYO = roll‐your‐own; FM = factory‐made. Model 1 reflects the ARIMAX model predicting smoking prevalence, model 2 reflects the ARIMAX model predicting quit attempt prevalence and model 3 reflects the ARIMAX model predicting quit success prevalence.

**TABLE 3:** Association between tax increases and per‐capita self‐reported cigarette consumption and average cost per FM and RYO cigarettes (adjusted for all tax policies, mass media and tobacco control policies)

	Model 4: per‐capita self‐reported cigarette consumption				Model 5: average cost per FM cigarette				Model 6: average cost per RYO			
	B	95% CI		P	B	95% CI		P	B	95% CI		P
		Lower	Upper			Lower	Upper			Lower	Upper	
Step level change 1 for FM (March 2010–December 2012)	−5.2	−14.6	5.3	0.318	0.6	−5.9	7.5	0.864	5.6	−3.1	15.0	0.218
Step level change 2 for FM (March 2012–December 2019)	−6.0	−15.4	4.4	0.247	3.7	−3.0	10.9	0.285	−0.4	−8.6	8.7	0.935
Pulse effect for RYO (March 2011)	−20.7	−32.4	−7.0	0.004	−1.0	−10.3	9.3	0.842	17.0	−1.4	38.9	0.072
Pulse effect for RYO (March 2016)	9.2	−6.9	28.1	0.280	−5.9	−13.3	2.1	0.145	3.2	−13.0	22.6	0.717
Pulse effect for RYO (November 2017)	−1.4	−15.9	15.7	0.866	9.8	−0.8	21.5	0.071	−1.3	−16.8	17.2	0.882
Pulse effect for RYO (November 2018)	3.5	−11.9	21.5	0.678	−3.1	−12.4	7.1	0.537	−20.7	−33.3	−5.8	0.008
Model specification	ARIMAX (0,1,1)				ARIMAX (0,1,1)				ARIMAX (0,1,1)			
AR												
MA	−0.546	−0.581	−0.507	< 0.001	−0.783	−0.904	−0.662	< 0.001	−1.139	−1.224	−1.053	< 0.001
SAR												
SMA												

AR = autoregressive; MA = moving average; SAR = seasonal autoregressive; SMA = seasonal moving average; ARIMAX = autoregressive integrated moving average with explanatory variable (ARIMAX), RYO = roll‐your‐own; FM = factory‐made. Model 4 reflects the ARIMAX model predicting per‐capita self‐reported cigarette consumption, model 5 reflects the ARIMAX model predicting average cost per FM cigarette and model 6 reflects the ARIMAX model predicting average cost per RYO cigarette.

A pulse in the rise in tax by 3% above inflation on RYO in November 2018 was also associated with the average cost per RYO cigarette (see Table 3). Paradoxically, the tax rise was associated with a 20.7% decline in the cost per RYO cigarette. Fitted values from the model indicated that this equated to a decrease from £0.15 in the month before to £0.12 in November 2018.

Supporting information, Table S3 shows the results for the unadjusted models and Supporting information, Table S4 shows the results for the models adjusted for all other tax increases. Bayes factors indicated that the data were largely insensitive to detect changes for the non‐significant findings across the outcome measures of interest (see Supporting information, Table S5).

#### Stratification by SES

The significant association remained between the pulse of the 10% rise in tax on RYO cigarettes and smoking prevalence and per‐capita self‐reported cigarette consumption among those classed as lower SES but not those of higher SES (Supporting information, Tables S6 and S7). The significant association remained between the pulse of the rise in tax by 3% above inflation on RYO in November 2018 and the cost per RYO cigarette only among those of lower SES. Surprisingly, there was also a decline in quit success rates among those of higher SES in March 2011 associated with the tax increase on RYO cigarettes by 10%, although the effect size was significantly elevated compared to the unstratified analyses, suggesting some model instability.

#### Stratification by predominant RYO versus FM use for smoking prevalence

Following stratification, the significant negative association remained between the pulse for the 10% rise in RYO‐targeted tax and smoking prevalence of both predominant FM and RYO cigarettes. A significant positive association was detected between the pulse in November 2018 for the tax rise on RYO and smoking prevalence of predominant FM smoked cigarettes (see Supporting information, Table S8).

#### Stratification by exclusive RYO and FM use for per‐capita self‐reported cigarette consumption

There was a significant association between the pulse of the rise in tax by 5% above inflation on RYO cigarettes in March 2016 and the average RYO cigarette consumption per day. During March 2016 the rise in tax was associated with a 29.4% increase in per‐capita self‐reported cigarette consumption of RYO cigarettes. Fitted values from the model indicated that this equated to increasing the average number of RYO cigarettes smoked from one per day in February 2016 to 1.30 per day in March 2016 (see Supporting information, Table S11).

#### Modelling significant associations using quarterly data

When re‐running the significant associations identified in the primary analysis using quarterly rather than monthly data, no statistically significant associations were found between the pulses of the rise in tax on RYO during the first quarter of 2011 and the cost per RYO cigarettes and per‐capita self‐reported cigarette consumption. The association between the pulse in the first quarter of 2011 for the tax rise on RYO and smoking prevalence was of a similar magnitude to the primary analysis using monthly data (−18.9 versus −21.1), although not significant. Bayes factors confirmed that the data were insensitive (see Supporting information, Table S12).

### Effect of FM‐targeted tax increases (step level effects)

#### Primary analysis

The step level change reflecting the rise in tax above inflation for FM cigarettes from March 2012 was associated with a 33.1% increase in the quit success rate (see Table 2). Fitted values from the model suggested that the rise in tax above inflation increased the success rate of quit attempts from an average of 13.9% before March 2012 to 16.6% after March 2012.

Supporting information, Table S3 shows the results for the unadjusted models and Supporting information, Table S4 shows the results for the models adjusted for all other tax increases. Bayes factors indicated that the data were largely insensitive to detect changes for the non‐significant findings across the outcome measures of interest (see Supporting information, Table S5).

#### Stratification by SES

Following stratification, the step level increase reflecting the rise in tax above inflation for FM cigarettes in March 2012 was associated with 19.0% increase in the quit success rate only among those of higher SES (see Supporting information, Tables S6 and S7).

#### Stratification by predominant RYO versus FM use for smoking prevalence

The above inflation tax on FM cigarettes initiated in March 2010 was associated with a step level 12.7% decline in smoking prevalence of predominant RYO cigarettes (see Supporting information, Table S8).

#### Stratification by exclusive RYO and FM use for per‐capita self‐reported cigarette consumption

No statistically significant associations were found between tax increases and average consumption per day of FM cigarettes after stratification by exclusive RYO and exclusive FM use (see Supporting information, Table S11).

#### Modelling significant associations using quarterly data

When re‐running the significant associations identified in the primary analysis using quarterly rather than monthly data, a step‐level change from the first quarter of 2012 to the last quarter of 2019, reflecting the rise in tax on FM cigarettes, was positively associated with the success of quit attempts (see Supporting information, Table S12).

## DISCUSSION

### Summary

The announcement of large tax increases on RYO cigarettes in England appear to have been associated with a temporary reduction in smoking prevalence, in mean cigarette consumption per day and, perhaps surprisingly, in the amount spent per RYO cigarette. These associations were only present among those of lower SES after stratification. The announcement of tax increases on FM cigarettes in England also appeared to be associated with a sustained increase in the prevalence of successful quit attempts. This association was only present among those of higher SES after stratification. However, some of the findings were sensitive to model specification and temporally specific. Bayes factors also indicated that our data were insensitive to detect other associations.

### Limitations

This study had several limitations. First, it was unexpected to find that the largest increase in tax to 5% above inflation from March 2012 to February 2013 was associated with a step level increase in quit success rates. Although we adjusted for mass media spend and tobacco control policies, other population level factors may account for this association, including the rise in use of e‐cigarettes in 2012 [23]. Secondly, the effect sizes for the pulse effects and confidence intervals are relatively large. The plausibility of these effects occurring during a 1‐month period as a consequence of announcing the tax increases needs to be considered. However, the analysis allowed for pulse effects to decay gradually or to decay abruptly and quarterly data were used in a sensitivity analysis. Thirdly, the STS requires participants to recall quit attempts in the past 12 months which could have introduced bias, although we have no reason to believe that reporting would differ over time. The STS also requires participants to recall their cigarette consumption and amount spent on cigarettes. STS estimates of cigarettes per day has been shown to align with sales data [12]. Fourthly, the findings might not generalize to other countries. Finally, the impact of several population‐level polices were only modelled as pulse effects. It is possible that findings might be different if a more comprehensive evaluation had been undertaken; for example, a consideration of step‐level changes or permanent changes. This was not possible with the number of different factors included in the models in the current study, but warrants further investigation. This study could also not adjust for several important policies, including the introduction and rise in MET, which creates a minimum price and discourages the selling of cheaper FM products. This occurred during the same month as the tax increases being investigated (November 2017), and therefore it was possible to disentangle effects. The MET has been shown to be effective in reducing tobacco industry revenues [3].

### Comparison with previous studies

In line with previous studies, the finding of a decrease in smoking prevalence following the accouncement of tax increases on RYO cigarettes suggests that this may be an effective intervention for reducing tobacco use [5, 6, 9, 10]. The fact that this effect was only present among those of lower SES indicates that this may be one strategy to reduce widening of social inequalities in health. Previous studies also report that smokers in lower SES groups are the most responsive to tobacco price increases [5], with RYO cigarettes being more common among those from disadvantaged backgrounds [24].

The finding of an increase in quit success as a function of increasing tax on FM cigarettes is also in line with previous studies [25, 26], as is the increased strength of this association among those of higher SES. Although Wilkinson *et al*. [9] found that those of lower SES had a larger immediate reduction in smoking prevalence in response to the 2010 tax than the higher SES group, it was not sustained. A possible explanation is a greater incidence of relapse among the lower SES group. Smokers in lower SES groups are generally more vulnerable to relapse due to their higher nicotine dependence and decreased quitting self‐efficacy [27]. More disadvantaged smokers also have a lower level of FM use and are therefore less affected by FM tax increases [24].

This study also found a reduction in the amount paid per RYO as a function of tax increases on RYO cigarettes—a finding which was also concentrated among those of lower SES. The lower price paid may be explained by users of more expensive RYO cigarettes being pushed back to FM cigarettes, leaving users of less expensive RYO, or simply RYO users switching to cheaper RYO products. There was evidence of switching behaviours in the sensitivity analysis. For example, the rise in tax on RYO in November 2018 was associated with a positive pulse effect in predominant FM smoking prevalence, suggesting a switch from RYO to FM cigarettes. Brand switching is a commonly used cost‐minimizing strategy by smokers [28].

The increased tax on RYO cigarettes was also associated with a reduction in per‐capita self‐reported cigarette consumption, predominantly among those of lower SES, which may be indicative of increased quitting activity and/or reduced uptake as a response to anticipation of the increased price of cigarettes. The latter would be consistent with the finding of a reduced smoking prevalence but not increased quit success rate among those of lower SES in response to tax rises. A surprising finding in the sensitivity analysis was that the 5% rise in tax on RYO in March 2016 was associated with an increase in per‐capita self‐reported cigarette consumption of predominant RYO cigarettes. This might reflect smokers pre‐empting the tax rise by reducing the amount of tobacco per cigarette, resulting in compensatory smoking. Although this is speculative, previous studies have reported a reduction in the weight of tobacco used in RYO cigarettes in response to price rises [15]. A decrease in the cost of RYO by the tobacco industry could also account for this finding. At the point when tobacco taxes are increased in March/April each year, the industry overshifts the tax increase on the more expensive brands, while absorbing the tax increase on lower price brands [29].

Bayes factors suggested that the failure to find an impact of smaller tax increases reflected data insensitivity, and the non‐significant results do not necessarily indicate no associations between these increases and key smoking outcomes. Some of the findings could also not be easily be explained and were unexpected. This introduces uncertainty about the findings from the models. For example, the above inflation tax on FM cigarettes initiated in March 2010 was associated with a step level 12.7% decline in smoking prevalence of predominant RYO cigarettes.

### Implications

This study found evidence for a positive impact of reasonably large increases in taxation, targeting FM and RYO cigarettes. Although we are cautious to infer cause and effect from this study, it lends support to the argument that the government should consider sizeable and sustained tax increases in future budgets, perhaps in line with the increases seen in other countries such as Australia (25%) [9]. We used the date of the announcement of the budget as the primary implementation date, and therefore any temporary pulse effects may reflect the announcement forewarning smokers about tax changes, while step level changes may reflect more of the impact of tax increases themselves. Anticipated future costs are one of the main determinants of current smoking [30]. Mass media campaigns are effective in reducing prevalence of smoking and thus it may be important to consider publicity around budget changes in the future [31].

This study also highlights the possible role that tobacco industry tactics may play in undermining tobacco increases (as evidenced by the associations with the cost of cigarettes) and strategies used by smokers to mitigate increased cost (as evidenced by possible switching between FM and RYO and from premium to non‐premium brands, and the reduction in consumption). However, tax rises nonetheless still had an impact on actual behaviour in the current study, in the form of increasing the rate of successful attempts to quit smoking.

## CONCLUSION

The announcement of the largest tax increases on cigarettes in England (5% on FM and 10% on RYO) appear to be associated with a temporary reduction in smoking prevalence, per‐capita self‐reported cigarette consumption but also with the cost paid per RYO cigarettes. They are also associated with a sustained increase in the success rate of quit attempts.

## DECLARATION OF INTERESTS

L.S. undertakes consultancy and research for and receives travel funds and hospitality from manufacturers of smoking cessation medications. E.B., J.B. and L.S. have received unrestricted research funding from Pfizer.

## AUTHOR CONTRIBUTIONS

**Emma Beard:** Conceptualization; formal analysis. **Jamie Brown:** Conceptualization. **Lion Shahab:** Conceptualization.

## Supporting information


**Table S1:** AIC values for original models and models including first order transfer functions to model gradual step level changes and decaying pulse effects
**Table S2:** Primary analyses – association between tax increases and prevalence of quit success and average cost per FM cigarette with abrupt temporary pulse effects and abrupt sustained step level changes for the tax increases on RYO and FM cigarettes (fully adjusted)
**Table S3:** Primary analyses – association between tax increases and overall smoking prevalence, prevalence of quit attempts, prevalence of quit success, average cigarette consumption and average cost per FM and RYO cigarettes (unadjusted)
**Table S4:** Primary analyses – association between tax increases and overall smoking prevalence, prevalence of quit attempts, prevalence of quit success, average cigarette consumption and average cost per FM and RYO cigarettes (adjusted for tax policies)
**Table S5:** Primary analyses – Bayes Factors for the fully adjusted models
**Table S6:** Sensitivity analysis Primary analyses – association between tax increases and overall smoking prevalence, prevalence of quit attempts, prevalence of quit success, average cigarette consumption and average cost per FM and RYO cigarettes for lower SES (fully adjusted)
**Table S7:** Sensitivity analysis Primary analyses – association between tax increases and overall smoking prevalence, prevalence of quit attempts, prevalence of quit success, average cigarette consumption and average cost per FM and RYO cigarettes for higher SES (fully adjusted)
**Table S8:** Sensitivity analyses – association between tax increases and overall smoking prevalence of predominant FM cigarettes and overall smoking prevalence of predominant RYO cigarettes (fully adjusted)
**Table S9:** Sensitivity analysis – association between tax increases and overall smoking prevalence, prevalence of quit attempts, prevalence of quit success, average cigarette consumption and average cost per FM and RYO cigarettes modelling the 5% increase in tax for FM cigarettes as a pulse effect (fully adjusted)
**Table S10:** Sensitivity analysis – association between tax increases and overall smoking prevalence, prevalence of quit attempts, prevalence of quit success, average cigarette consumption and average cost per FM and RYO cigarettes modelling the tax increases for RYO cigarettes as step level changes (fully adjusted)
**Table S11:** Sensitivity analysis – association between tax increases and overall average cigarette consumption stratified by exclusive FM and RYO cigarettes (fully adjusted)
**Table S12:** Association between tax increases and overall smoking prevalence and prevalence of quit success using quarterly data (fully adjusted)
