# Supplementary material for: Smoking prevalence following the announcement of tobacco tax increases in England between 2007 and 2019: an interrupted time–series analysis
Source: Addiction. 2022 May 1;117(9):2481–92. doi: 10.1111/add.15898 (PMC9545480; doi:10.1111/add.15898)
Supplement: Supplementary file 1 — Table S1: AIC values for original models and models including first order transfer functions to model gradual step level changes and decaying pulse effects Table S2: Primary analyses – association between tax increases and prevalence of quit success and average cost per FM cigarette with abrupt temporary pulse effects and abrupt sustained step level changes for the tax increases on RYO and FM cigarettes (fully adjusted) Table S3: Primary analyses – association between tax increases and overall smoking prevalence, prevalence of quit attempts, prevalence of quit success, average cigarette consumption and average cost per FM and RYO cigarettes (unadjusted) Table S4: Primary analyses – association between tax increases and overall smoking prevalence, prevalence of quit attempts, prevalence of quit success, average cigarette consumption and average cost per FM and RYO cigarettes (adjusted for tax policies) Table S5: Primary analyses – Bayes Factors for the fully adjusted models Table S6: Sensitivity analysis Primary analyses – association between tax increases and overall smoking prevalence, prevalence of quit attempts, prevalence of quit success, average cigarette consumption and average cost per FM and RYO cigarettes for lower SES (fully adjusted) Table S7: Sensitivity analysis Primary analyses – association between tax increases and overall smoking prevalence, prevalence of quit attempts, prevalence of quit success, average cigarette consumption and average cost per FM and RYO cigarettes for higher SES (fully adjusted) Table S8: Sensitivity analyses – association between tax increases and overall smoking prevalence of predominant FM cigarettes and overall smoking prevalence of predominant RYO cigarettes (fully adjusted) Table S9: Sensitivity analysis – association between tax increases and overall smoking prevalence, prevalence of quit attempts, prevalence of quit success, average cigarette consumption and average cost per FM and RYO cigarettes modelling the 5% increa [file ADD-117-2481-s001.docx]

**Supplementary material**

Supplementary Table 1: AIC values for original models and models including first order transfer functions to model gradual step level changes and decaying pulse effects

|  | Smoking prevalence | Quit attempt prevalence | Quit success prevalence | Average RYO cigarette consumption | Average cost per FM cigarette | Average cost per RYO cigarette |
| --- | --- | --- | --- | --- | --- | --- |
| Abrupt sustained step level change (zero order transfer function original model) | -377 | -254 | 43 | -300 | -412 | -266 |
| Gradual sustained step level change (first order transfer function) | -374 | -252 | 44 | -302 | -407 | -264 |
| Abrupt temporary pulse effect (zero order transfer function original model) | -377 | -254 | 43 | -300 | -412 | -266 |
| Temporary pulse effect which decays over time (first order transfer function) | -374 | -251 | 40 | -297 | -405 | -265 |

Supplementary Table 2: Primary analyses – association between tax increases and prevalence of quit success and average cost per FM cigarette with abrupt temporary pulse effects and abrupt sustained step level changes for the tax increases on RYO and FM cigarettes (fully adjusted)

|  | Model 1: Quit success prevalence | | | | Models 2: Average cost per FM cigarette | | | |
| --- | --- | --- | --- | --- | --- | --- | --- | --- |
|  | B | 95%CI | |  | B | 95%CI | | p |
|  |  | Lower | Upper | p |  | Lower | Upper |  |
| Step level change 1 for FM (March 2010 – December 2012) | -15.4 | -35.3 | 10.6 | 0.222 | 0.6 | -6.0 | 7.6 | 0.866 |
| Step level change 2 for FM (March 2012 – December 2019) | 22.1 | -4.1 | 55.4 | 0.106 | 3.9 | -2.9 | 11.1 | 0.269 |
| Pulse effect for RYO (March 2011) | -31.1 | -58.4 | 14.0 | 0.147 | -1.0 | -10.5 | 9.5 | 0.850 |
| Pulse effect for RYO (March 2016) | 7.6 | -35.2 | 78.6 | 0.778 | -5.6 | -14.8 | 4.5 | 0.264 |
| Pulse effect for RYO (November 2017) | -12.5 | -47.1 | 44.6 | 0.602 | 9.5 | -1.0 | 21.2 | 0.078 |
| Pulse effect for RYO (November 2018) | -16.8 | -49.9 | 38.1 | 0.477 | -3.1 | -12.5 | 7.3 | 0.544 |
| Model specification  AR  MA  SAR  SMA | ARIMAX(0,1,1)_12_  -1.082 | -1.204 | -0.960 | <0.001 | ARIMAX(0,1,1)_12_  -0.783 | -0.906 | -0.660 | <0.001 |

Supplementary Table 3: Primary analyses – association between tax increases and overall smoking prevalence, prevalence of quit attempts, prevalence of quit success, average cigarette consumption and average cost per FM and RYO cigarettes (unadjusted)

|  | Models 1-6: Smoking prevalence | | | | Models 7-12: Quit attempt prevalence | | | | Models 13-18: Quit success prevalence | | | |
| --- | --- | --- | --- | --- | --- | --- | --- | --- | --- | --- | --- | --- |
|  | B | 95%CI | | P | B | 95%CI |  | P | B | 95%CI |  | p |
|  |  | Lower | Upper |  |  | Lower | Upper |  |  | Lower | Upper |  |
| Step level change 1 for FM (March 2010 – December 2012) | -3.6 | -11.2 | 4.7 | 0.384 | -2.0 | -13.1 | 14.6 | 0.979 | -23.5 | -42.1 | 1.1 | 0.059 |
| Step level change 2 for FM (March 2012 – December 2019) | -2.4 | -10.1 | 6.0 | 0.569 | 9.7 | -4.3 | 25.8 | 0.184 | 20.5 | -3.5 | 50.5 | 0.100 |
| Pulse effect for RYO (March 2011) | -21.0 | -30.4 | -10.3 | <0.001 | -3.5 | -19.8 | 16.2 | 0.710 | -35.7 | -61.4 | 7.1 | 0.090 |
| Pulse effect for RYO (March 2016) | 3.3 | -9.5 | 17.9 | 0.630 | -10.8 | -25.8 | 7.3 | 0.225 | 6.7 | -3.6 | 18.3 | 0.214 |
| Pulse effect for RYO (November 2017) | 1.3 | -11.3 | 15.6 | 0.852 | -6.4 | -22.3 | 12.8 | 0.488 | -1.6 | -4.0 | 47.4 | 0.637 |
| Pulse effect for RYO (November 2018) | 6.9 | -6.3 | 22.0 | 0.322 | 3.3 | -14.1 | 24.3 | 0.729 | -19.8 | -51.3 | 32.3 | 0.385 |
| Model specification | ARIMAX(0,1,1)_12_ |  |  |  | ARIMAX(0,1,1)_12_ | | | | ARIMAX(0,1,1)_12_ | | | |

Supplementary Table 3 (continued): Primary analyses – association between tax increases and overall smoking prevalence, prevalence of quit attempts, prevalence of quit success prevalence, average cigarette consumption and average cost per FM and RYO cigarettes (unadjusted)

|  | Models 19-24: Average cigarette consumption | | | | Models 25-30: Average cost per FM cigarette | | | | Models 31-36: Average cost per RYO cigarette | | | |
| --- | --- | --- | --- | --- | --- | --- | --- | --- | --- | --- | --- | --- |
|  | B | 95%CI | | P | B | 95%CI |  | P | B | 95%CI |  | p |
|  |  | Lower | Upper |  |  | Lower | Upper |  |  | Lower | Upper |  |
| Step level change 1 for FM (March 2010 – December 2012) | -4.6 | -14.5 | 6.4 | 0.395 | -0.9 | -6.7 | 6.8 | 0.958 | 7.0 | -1.9 | 16.8 | 0.127 |
| Step level change 2 for FM (March 2012 – December 2019) | -5.5 | -15.3 | 5.3 | 0.303 | 4.3 | -2.2 | 11.4 | 0.201 | -0.6 | -9.3 | 9.1 | 0.906 |
| Pulse effect for RYO (March 2011) | -20.5 | -32.3 | -6.6 | 0.005 | -1.2 | -11.0 | 9.6 | 0.814 | 18.1 | -1.1 | 41.0 | 0.065 |
| Pulse effect for RYO (March 2016) | 8.5 | -8.0 | 27.9 | 0.335 | -5.3 | -14.7 | 5.1 | 0.307 | 2.5 | -14.4 | 22.6 | 0.791 |
| Pulse effect for RYO (November 2017) | -2.2 | -17.1 | 15.3 | 0.788 | 10.3 | -0.4 | 22.3 | 0.060 | -2.0 | -18.0 | 17.3 | 0.829 |
| Pulse effect for RYO (November 2018) | 4.7 | -11.2 | 23.5 | 0.584 | -4.1 | -13.6 | 6.4 | 0.428 | -20.0 | -32.9 | -4.7 | 0.013 |
| Model specification | ARIMAX(0,1,1)_12_ |  |  |  | ARIMAX(0,1,1)_12_ | | | | ARIMAX(0,1,1)_12_ | | | |

Supplementary Table 4: Primary analyses – association between tax increases and overall smoking prevalence, prevalence of quit attempts, prevalence of quit success, , average cigarette consumption and average cost per FM and RYO cigarettes (adjusted for tax policies)

|  | Models 1: Smoking prevalence | | | | Models 2: Quit attempt prevalence | | | | Models 3: Quit success prevalence | | | |  |  |
| --- | --- | --- | --- | --- | --- | --- | --- | --- | --- | --- | --- | --- | --- | --- |
|  | B | 95%CI | | P | B | 95%CI |  | P | B | 95%CI |  | p |  |  |
|  |  | Lower | Upper |  |  | Lower | Upper |  |  | Lower | Upper |  |  |  |
| Step level change 1 for FM (March 2010 – December 2012) | -3.2 | -10.7 | 4.7 | 0.413 | -0.2 | -13.0 | 14.6 | 0.979 | -18.8 | -36.8 | 4.4 | 0.104 |  |  |
| Step level change 2 for FM (March 2012 – December 2019) | -2.7 | -10.2 | 5.5 | 0.520 | 9.8 | -4.3 | 26.0 | 0.184 | 20.1 | -6.2 | 53.7 | 0.147 |  |  |
| Pulse effect for RYO (March 2011) | -21.0 | -30.4 | -10.4 | <0.001 | -3.3 | -19.4 | 16.1 | 0.717 | -32.0 | -58.2 | 10.8 | 0.122 |  |  |
| Pulse effect for RYO (March 2016) | 3.3 | -9.0 | 17.1 | 0.615 | -10.8 | -25.6 | 7.1 | 0.221 | 7.7 | -2.7 | 19.0 | 0.152 |  |  |
| Pulse effect for RYO (November 2017) | 1.3 | -10.7 | 14.9 | 0.838 | -6.3 | -22.1 | 22.0 | 0.487 | -20.8 | -51.5 | 29.4 | 0.352 |  |  |
| Pulse effect for RYO (November 2018) | 6.9 | -5.7 | 21.3 | 0.298 | -3.3 | -13.9 | 24.0 | 0.726 | -29.0 | -56.5 | 16.0 | 0.172 |  |  |
| Model specification  AR  MA  SAR  SMA | ARIMAX(0,1,1)_12_  -0.801 | -0.890 | -0.712 | <0.001 | ARIMAX(0,1,1)_12_  -0.717 | -0.841 | -0.592 | <0.001 | ARIMAX(0,1,1)_12_  -0.907 | -0.998 | -0.817 | <0.001 |  |  |

Supplementary Table 4 (continued): Primary analyses – association between tax increases and overall smoking prevalence, prevalence of quit attempts, prevalence of quit success, average cigarette consumption and average cost per FM and RYO cigarettes (adjusted for tax policies)

|  | Models 4: Average cigarette consumption | | | | Models 5: Average cost per FM cigarette | | | | Models 6: Average cost per RYO cigarette | | | |
| --- | --- | --- | --- | --- | --- | --- | --- | --- | --- | --- | --- | --- |
|  | B | 95%CI | | P | B | 95%CI |  | P | B | 95%CI |  | p |
|  |  | Lower | Upper |  |  | Lower | Upper |  |  | Lower | Upper |  |
| Step level change 1 for FM (March 2010 – December 2012) | -4.4 | -13.7 | 6.0 | 0.391 | -0.2 | -6.5 | 6.5 | 0.957 | 6.5 | -2.5 | 16.4 | 0.163 |
| Step level change 2 for FM (March 2012 – December 2019) | -6.4 | -15.7 | 3.9 | 0.213 | 4.2 | -2.4 | 11.4 | 0.220 | -1.0 | -9.5 | 8.2 | 0.820 |
| Pulse effect for RYO (March 2011) | -20.6 | -32.4 | -6.8 | 0.005 | -1.1 | -10.7 | 9.5 | 0.835 | 17.4 | -1.1 | 39.3 | 0.067 |
| Pulse effect for RYO (March 2016) | 8.8 | -7.3 | 27.7 | 0.301 | -5.2 | -14.5 | 5.1 | 0.308 | 2.9 | -13.3 | 22.1 | 0.743 |
| Pulse effect for RYO (November 2017) | -2.1 | -16.6 | 14.8 | 0.792 | 10.3 | -0.4 | 22.1 | 0.059 | -2.2 | -17.6 | 16.0 | 0.799 |
| Pulse effect for RYO (November 2018) | 4.6 | -10.9 | 22.7 | 0.581 | -4.0 | -13.3 | 6.3 | 0.431 | -19.9 | -32.6 | -5.0 | 0.011 |
| Model specification  AR  MA  SAR  SMA | ARIMAX(0,1,1)_12_  -0.794 | -0.875 | -0.713 | <0.001 | ARIMAX(0,1,1)_12_  -0.796 -0.914 -0.677 <0.001 | | | | ARIMAX(0,1,1)_12_  -1.155 -1.245 -1.064 <0.001 | | | |

Supplementary Table 5: Primary analyses – Bayes Factors for the fully adjusted models

|  | Smoking prevalence  BF(Robustness region) | Quit attempt prevalence  BF(Robustness region) | Quit success prevalence  BF(Robustness region) | Average cigarette consumption  BF(Robustness region) | Average cost per FM cigarette  BF(Robustness region) | Average cost per RYO cigarette BF(Robustness region) |
| --- | --- | --- | --- | --- | --- | --- |
| Step level change 1 for FM (March 2010 – December 2012) | 1.49 (0 to 34.5) | 0.87 (0 to 20.5) | 0.47 (0 to 7.0) | 1.43 (0 to 42.5) | 0.57 (0 to 7.9) | 0.42 (0 to 5.8) |
| Step level change 2 for FM (March 2012 – December 2019) | 1.10 (0 to 23.2) | 1.53 (0 to 91.5) | 11.57 (2.2 to $\infty$)* | 0.82 (0 to 16.8) | 0.38 (0 to 4.8) | 0.76 (0 to 13.5) |
| Pulse effect for RYO (March 2011) | 57.8 (1.4 to $\infty$)* | 0.81 (0 to 19.0) | 0.73 (0 to 18.0) | 7.30 (2.4 to $\infty$)* | 0.85 (0 to 17.2) | 0.60 (0 to 10.7) |
| Pulse effect for RYO (March 2016) | 1.02 (0 to 27.7) | 0.59 (0 to 10.0) | 1.69 (0 to 75.0) | 0.66 (0 to 12.2) | 2.15 (0 to 66.5) | 0.81 (0 to 19.9) |
| Pulse effect for RYO (November 2017) | 0.73 (0 to 14.5) | 0.76 (o to 16.7) | 0.81 (0 to 25.8) | 0.95 (0 to 27.2) | 0.41 (0 to 5.8) | 0.94 (0 to 28.2) |
| Pulse effect for RYO (November 2018) | 0.61 (0 to 10.4) | 1.05 (0 to 39.5) | 0.65 (0 to 13.2) | 0.78 (0 to 17.5) | 1.09 (0 to 26.2) | 5.06 (2.9 to $\infty$)* |

Note: BF=Bayes Factor; * Evidence for the alternative hypothesis of an impact

Supplementary Table 6: Sensitivity analysis Primary analyses – association between tax increases and overall smoking prevalence, prevalence of quit attempts, prevalence of quit success, average cigarette consumption and average cost per FM and RYO cigarettes for lower SES (fully adjusted)

|  | Models 1: Smoking prevalence | | | | Models 2: Quit attempt prevalence | | | | Models 3: Quit success prevalence | | | |  |  |
| --- | --- | --- | --- | --- | --- | --- | --- | --- | --- | --- | --- | --- | --- | --- |
|  | B | 95%CI | | P | B | 95%CI |  | P | B | 95%CI |  | P |  |  |
|  |  | Lower | Upper |  |  | Lower | Upper |  |  | Lower | Upper |  |  |  |
| Step level change 1 for FM (March 2010 – December 2012) | -1.8 | -9.6 | 6.6 | 0.662 | -2.8 | -16.6 | 10.4 | 0.718 | -28.7 | -54.8 | 12.6 | 0.147 |  |  |
| Step level change 2 for FM (March 2012 – December 2019) | -4.4 | -11.9 | 3.8 | 0.279 | 5.3 | -9.6 | 13.3 | 0.504 | 21.5 | -22.0 | 89.5 | 0.390 |  |  |
| Pulse effect for RYO (March 2011) | -25.8 | -36.0 | -13.9 | <0.001 | -15.4 | -32.8 | 22.9 | 0.156 | 12.4 | -53.5 | 171.8 | 0.795 |  |  |
| Pulse effect for RYO (March 2016) | 1.3 | -12.6 | 17.6 | 0.863 | -8.1 | -27.1 | 6.6 | 0.477 | 29.0 | -46.6 | 211.4 | 0.571 |  |  |
| Pulse effect for RYO (November 2017) | 2.2 | -11.9 | 18.6 | 0.770 | 2.9 | -18.5 | 16.0 | 0.809 | 1.6 | -58.0 | 145.5 | 0.972 |  |  |
| Pulse effect for RYO (November 2018) | 7.5 | -7.4 | 24.9 | 0.343 | 1.4 | -19.7 | 30.0 | 0.908 | 30.7 | -46.3 | 218.0 | 0.554 |  |  |
| Model specification  AR  MA  SAR  SMA | ARIMAX(0,1,1)_12_  -0.852 | -0.928 | -0.776 | <0.001 | ARIMAX(0,1,1)_12_  -0.784 | -0.897 | -0.671 | <0.001 | ARIMAX(0,1,1)_12_  -0.900 | -0.993 | -0.807 | <0.001 |  |  |

Supplementary Table 6 (continued): Sensitivity analysis Primary analyses – association between tax increases and overall smoking prevalence, prevalence of quit attempts, prevalence of quit success, average cigarette consumption and average cost per FM and RYO cigarettes for lower SES (fully adjusted)

|  | Models 4: Average cigarette consumption | | | | Models 5: Average cost per FM cigarette | | | | Models 6: Average cost per RYO cigarette | | | |
| --- | --- | --- | --- | --- | --- | --- | --- | --- | --- | --- | --- | --- |
|  | B | 95%CI | | P | B | 95%CI |  | P | B | 95%CI |  | P |
|  |  | Lower | Upper |  |  | Lower | Upper |  |  | Lower | Upper |  |
| Step level change 1 for FM (March 2010 – December 2012) | -4.7 | -15.0 | 7.0 | 0.416 | 0.6 | -5.4 | 7.0 | 0.849 | 3.8 | -5.2 | 13.7 | 0.415 |
| Step level change 2 for FM (March 2012 – December 2019) | -7.1 | -17.2 | 4.2 | 0.210 | 5.1 | -1.4 | 11.9 | 0.126 | 1.2 | -7.5 | 10.7 | 0.793 |
| Pulse effect for RYO (March 2011) | -26.0 | -39.0 | -10.3 | 0.002 | 1.1 | -10.3 | 14.0 | 0.854 | 16.8 | -1.4 | 38.3 | 0.072 |
| Pulse effect for RYO (March 2016) | 8.2 | -10.8 | 31.3 | 0.422 | -9.3 | -19.6 | 2.3 | 0.111 | 8.4 | -8.4 | 28.4 | 0.347 |
| Pulse effect for RYO (November 2017) | -2.6 | -19.7 | 18.2 | 0.791 | 9.7 | -2.7 | 23.8 | 0.131 | 0.5 | -15.1 | 19.1 | 0.952 |
| Pulse effect for RYO (November 2018) | 5.6 | -13.1 | 28.2 | 0.584 | -6.4 | -17.0 | 5.6 | 0.282 | -18.6 | -31.4 | -3.5 | 0.018 |
| Model specification  AR  MA  SAR  SMA | ARIMAX(0,1,1)_12_  -0.828 | -0.911 | -0.744 | <0.001 | ARIMAX(0,1,1)_12_  -1.143 -1.245 -1.041 <0.001 | | | | ARIMAX(0,1,1)_12_  -1.162 -1.259 -1.061 <0.001 | | | |

Supplementary Table 7: Sensitivity analysis Primary analyses – association between tax increases and overall smoking prevalence, prevalence of quit attempts, prevalence of quit success, average cigarette consumption and average cost per FM and RYO cigarettes for higher SES (fully adjusted)

|  | Models 1: Smoking prevalence | | | | Models 2: Quit attempt prevalence | | | | Models 3: Quit success prevalence | | | |  |  |
| --- | --- | --- | --- | --- | --- | --- | --- | --- | --- | --- | --- | --- | --- | --- |
|  | B | 95%CI | | P | B | 95%CI |  | P | B | 95%CI |  | P |  |  |
|  |  | Lower | Upper |  |  | Lower | Upper |  |  | Lower | Upper |  |  |  |
| Step level change 1 for FM (March 2010 – December 2012) | -7.6 | -18.1 | 4.3 | 0.198 | 2.4 | -12.5 | 20.1 | 0.776 | 0.6 | -15.5 | 19.8 | 0.945 |  |  |
| Step level change 2 for FM (March 2012 – December 2019) | -1.2 | -12.5 | 11.7 | 0.850 | 13.7 | -2.6 | 32.6 | 0.103 | 19.0 | 2.6 | 38.0 | 0.021 |  |  |
| Pulse effect for RYO (March 2011) | -14.5 | -28.8 | 2.6 | 0.092 | 13.0 | -12.1 | 45.1 | 0.340 | -58.3 | -78.2 | -20.4 | 0.008 |  |  |
| Pulse effect for RYO (March 2016) | 9.3 | -8.9 | 31.3 | 0.338 | -15.0 | -33.8 | 9.2 | 0.203 | -7.7 | -51.2 | 74.5 | 0.806 |  |  |
| Pulse effect for RYO (November 2017) | 2.4 | -14.7 | 23.0 | 0.797 | -15.0 | -34.0 | 9.4 | 0.206 | -21.1 | -58.2 | 49.0 | 0.466 |  |  |
| Pulse effect for RYO (November 2018) | 3.8 | -13.7 | 24.9 | 0.690 | 8.8 | -15.5 | 39.9 | 0.516 | -43.3 | -70.2 | 8.2 | 0.085 |  |  |
| Model specification  AR  MA  SAR  SMA | ARIMAX(0,1,1)_12_  -0.794 | -0.888 | -0.700 | <0.001 | ARIMAX(0,1,1)_12_  -0.815 | -0.920 | -0.710 | <0.001 | ARIMAX(0,1,1)_12_  -1.000 | -1.034 | -0.966 | <0.001 |  |  |

Supplementary Table 7 (continued): Sensitivity analysis Primary analyses – association between tax increases and overall smoking prevalence, prevalence of quit attempts, prevalence of quit success, average cigarette consumption and average cost per FM and RYO cigarettes for higher SES (fully adjusted)

|  | Models 4: Average cigarette consumption | | | | Models 5: Average cost per FM cigarette | | | | Models 6: Average cost per RYO cigarette | | | |
| --- | --- | --- | --- | --- | --- | --- | --- | --- | --- | --- | --- | --- |
|  | B | 95%CI | | P | B | 95%CI |  | P | B | 95%CI |  | P |
|  |  | Lower | Upper |  |  | Lower | Upper |  |  | Lower | Upper |  |
| Step level change 1 for FM (March 2010 – December 2012) | -6.6 | -19.2 | 7.9 | 0.354 | 1.89 | -6.7 | 11.3 | 0.683 | 8.6 | -3.2 | 21.8 | 0.160 |
| Step level change 2 for FM (March 2012 – December 2019) | -6.0 | -18.8 | 8.8 | 0.407 | 4.7 | -4.2 | 14.4 | 0.313 | 2.7 | -9.6 | 16.7 | 0.680 |
| Pulse effect for RYO (March 2011) | -12.1 | -29.8 | 10.1 | 0.261 | -3.4 | -16.7 | 12.0 | 0.650 | 20.7 | -13.3 | 68.0 | 0.266 |
| Pulse effect for RYO (March 2016) | 13.10 | -9.7 | 41.6 | 0.284 | 5.4 | -9.1 | 22.3 | 0.486 | -9.8 | -35.0 | 25.2 | 0.538 |
| Pulse effect for RYO (November 2017) | 1.9 | -18.7 | 27.7 | 0.870 | 10.8 | -4.5 | 28.5 | 0.177 | -3.4 | -30.6 | 34.4 | 0.837 |
| Pulse effect for RYO (November 2018) | 0.6 | -19.8 | 26.2 | 0.968 | 5.3 | -9.3 | 22.2 | 0.500 | -26.5 | -47.2 | 2.3 | 0.068 |
| Model specification  AR  MA  SAR  SMA | ARIMAX(0,1,1)_12_  -0.799 | -0.882 | -0.713 | <0.001 | ARIMAX(0,1,1)_12_  -0.828 | -0.924 | -0.713 | <0.001 | ARIMAX(0,1,1)_12_  -1.053 | -0.849 | -0.992 | <0.001 |

| Supplementary Table 8: Sensitivity analyses – association between tax increases and overall smoking prevalence of predominant FM cigarettes and overall smoking prevalence of predominant RYO cigarettes (fully adjusted) | | | | | | | | |
| --- | --- | --- | --- | --- | --- | --- | --- | --- |
|  | Smoking prevalence of predominant RYO cigarettes | | | | Smoking prevalence of predominant FM cigarettes | | | |
|  | B | 95%CI | | P | B | 95%CI | | p |
|  |  | Lower | Upper |  |  | Lower | Upper |  |
| Step level change 1 for FM (March 2010 – December 2012) | -12.7 | -20.8 | -3.9 | 0.005 | -6.4 | -15.9 | 4.3 | 0.231 |
| Step level change 2 for FM (March 2012 – December 2019) | 3.7 | -6.7 | 15.0 | 0.503 | -100.0 | -16.6 | 4.7 | 0.246 |
| Pulse effect for RYO (March 2011) | -18.4 | -32.2 | -1.7 | 0.032 | -22.4 | -34.9 | -7.4 | 0.005 |
| Pulse effect for RYO (March 2016) | 1.9 | -15.4 | 22.6 | 0.844 | 5.3 | -11.7 | 25.6 | 0.559 |
| Pulse effect for RYO (November 2017) | -6.6 | -22.4 | 12.5 | 0.474 | 7.8 | -9.6 | 28.7 | 0.404 |
| Pulse effect for RYO (November 2018) | -13.0 | -27.7 | 4.8 | 0.142 | 20.9 | 1.3 | 44.3 | 0.036 |
| Model specification  AR  MA  SAR  SMA | ARIMAX(0,1,1)_12_  -1.150 | -1.290 | -1.011 | <0.001 | ARIMAX(0,1,1)_12_  -1.221 | -1.343 | -1.099 | <0.001 |

Supplementary Table 9: Sensitivity analysis – association between tax increases and overall smoking prevalence, prevalence of quit attempts, prevalence of quit success, average cigarette consumption and average cost per FM and RYO cigarettes modelling the 5% increase in tax for FM cigarettes as a pulse effect (fully adjusted)

|  | Models 1: Smoking prevalence | | | | Models 2: Quit attempt prevalence | | | | Models 3: Quit success prevalence | | | |  |  |
| --- | --- | --- | --- | --- | --- | --- | --- | --- | --- | --- | --- | --- | --- | --- |
|  | B | 95%CI | | P | B | 95%CI |  | P | B | 95%CI |  | p |  |  |
|  |  | Lower | Upper |  |  | Lower | Upper |  |  | Lower | Upper |  |  |  |
| Step level change 1 for FM (March 2010 – December 2012) | -4.2 | -11.8 | 8.8 | 0.306 | 0.2 | -12.9 | 15.3 | 0.978 | -21.1 | -40.9 | 5.3 | 0.108 |  |  |
| Pulse effect for FM (March 2012) | 5.8 | -6.7 | 4.0 | 0.380 | 7.5 | -10.6 | 29.0 | 0.444 | -36.0 | -61.1 | 5.0 | 0.077 |  |  |
| Pulse effect for RYO (March 2011) | -21.0 | -30.2 | 19.8 | <0.001 | -3.3 | -19.5 | 16.1 | 0.713 | -32.4 | -58.8 | 10.8 | 0.120 |  |  |
| Pulse effect for RYO (March 2016) | 4.0 | -8.1 | -10.6 | 0.540 | -11.0 | -20.5 | 6.9 | 0.215 | 4.4 | -36.4 | 71.1 | 0.866 |  |  |
| Pulse effect for RYO (November 2017) | 2.1 | -9.9 | 17.7 | 0.741 | -6.7 | -22.5 | 12.3 | 0.463 | -13.1 | -47.0 | 42.6 | 0.580 |  |  |
| Pulse effect for RYO (November 2018) | 5.8 | -6.7 | 15.7 | 0.377 | 4.0 | -13.7 | 25.1 | 0.683 | -14.9 | -48.2 | 39.9 | 0.525 |  |  |
| Model specification  AR  MA  SAR  SMA | ARIMAX(0,1,1)_12_  -0.789 | 0.877 | -0.701 | <0.001 | ARIMAX(0,1,1)_12_  -0.789 | 0.877 | -0.701 | <0.001 | ARIMAX(0,1,1)_12_  -0.873 | 0.953 | -0.793 | <0.001 |  |  |

Supplementary Table 9: Sensitivity analysis – association between tax increases and overall smoking prevalence, prevalence of quit attempts, prevalence of quit success, average cigarette consumption and average cost per FM and RYO cigarettes modelling the 5% increase in tax for FM cigarettes as a pulse effect (fully adjusted)

|  | Models 4: Average cigarette consumption | | | | Models 5: Average cost per FM cigarette | | | | Models 6: Average cost per RYO cigarette | | | |
| --- | --- | --- | --- | --- | --- | --- | --- | --- | --- | --- | --- | --- |
|  | B | 95%CI | | P | B | 95%CI |  | P | B | 95%CI |  | p |
|  |  | Lower | Upper |  |  | Lower | Upper |  |  | Lower | Upper |  |
| Step level change 1 for FM (March 2010 – December 2012) | -5.2 | -14.9 | 5.5 | 0.327 | 0.6 | -6.1 | 7.9 | 0.858 | 5.5 | -3.2 | 14.9 | 0.224 |
| Pulse effect for FM (March 2012) | 5.0 | -10.5 | 23.2 | 0.548 | 0.1 | -9.6 | 10.7 | 0.990 | 4.5 | -11.9 | 24.0 | 0.610 |
| Pulse effect for RYO (March 2011) | -20.5 | -32.2 | -6.7 | 0.005 | -1.1 | -10.6 | 9.4 | 0.830 | 17.1 | -1.2 | 39.9 | 0.069 |
| Pulse effect for RYO (March 2016) | 9.1 | -7.0 | 28.0 | 0.283 | -5.8 | -14.9 | 4.3 | 0.253 | 3.2 | -13.0 | 22.5 | 0.716 |
| Pulse effect for RYO (November 2017) | -1.3 | -15.9 | 15.7 | 0.869 | 9,6 | -1.0 | 21.2 | 0.078 | -1.3 | -16.8 | 17.2 | 0.885 |
| Pulse effect for RYO (November 2018) | 3.4 | -12.0 | 21.4 | 0.687 | -3.1 | -12.4 | 7.3 | 0.550 | -20.8 | -33.3 | -5.9 | 0.008 |
| Model specification  AR  MA  SAR  SMA | ARIMAX(0,1,1)_12_  -0.777 | -0.860 | 0.693 | <0.001 | ARIMAX(0,1,1)_12_  -0.770 -0.887 -0.652 <0.001 | | | | ARIMAX(0,1,1)_12_  -01.139 -1.224 -1.053 <0.001 | | | |

Supplementary Table 10: Sensitivity analysis – association between tax increases and overall smoking prevalence, prevalence of quit attempts, prevalence of quit success, average cigarette consumption and average cost per FM and RYO cigarettes modelling the tax increases for RYO cigarettes as step level changes (fully adjusted)

|  | Models 1: Smoking prevalence | | | | Models 2: Quit attempt prevalence | | | | Models 3: Quit success prevalence | | | |  |  |
| --- | --- | --- | --- | --- | --- | --- | --- | --- | --- | --- | --- | --- | --- | --- |
|  | B | 95%CI | | P | B | 95%CI |  | P | B | 95%CI |  | p |  |  |
|  |  | Lower | Upper |  |  | Lower | Upper |  |  | Lower | Upper |  |  |  |
| Step level change 1 for FM (March 2010 – December 2012) | -3.5 | -10.0 | 3.5 | 0.311 | -0.2 | -12.5 | 14.0 | 0.981 | -21.6 | -34.1 | -6.8 | 0.006 |  |  |
| Step level change 2 for FM (March 2012 – December 2019) | -2.5 | -9.0 | 4.5 | 0.479 | 9.7 | -3.8 | 25.4 | 0.168 | 21.0 | 3.1 | 42.2 | 0.020 |  |  |
| Step level change 1 RYO (March 2011-December 2019) | -5.5 | -12.0 | 1.5 | 0.120 | -2.3 | -14.5 | 11.7 | 0.735 | 26.0 | 2.9 | 54.0 | 0.025 |  |  |
| Step level change 2 RYO (March 2016-December 2019) | -4.2 | -10.9 | 2.9 | 0.242 | -3.1 | -15.0 | 10.7 | 0.652 | 3.8 | -9.4 | 18.9 | 0.593 |  |  |
| Step level change 3 RYO (November 2017-December 2019) | 0.1 | -6.9 | 7.4 | 0.990 | -8.7 | -20.1 | 4.3 | 0.181 | -12.5 | -27.5 | 5.8 | 0.169 |  |  |
| Step level change 4 RYO (November 2018-December 2019) | -9.0 | -15.1 | -2.5 | 0.008 | -0.9 | -13.2 | 13.2 | 0.896 | -11.8 | -27.3 | 6.9 | 0.201 |  |  |
| Model specification  AR  MA  SAR  SMA | ARIMAX(0,1,1)_12_  -0.865 | -0.951 | -0.780 | <0.001 | ARIMAX(0,1,1)_12_  -0.746 | -0.872 | -0.620 | <0.001 | ARIMAX(0,1,1)_12_  -1.000 | -1.034 | -0.966 | <0.001 |  |  |

Supplementary Table 10 (continued): Sensitivity analysis – association between tax increases and overall smoking prevalence, prevalence of quit attempts, prevalence of quit success, average cigarette consumption and average cost per FM and RYO cigarettes modelling the tax increases for RYO cigarettes as step level changes (fully adjusted)

|  | Models 4: Average cigarette consumption | | | | Models 5: Average cost per FM cigarette | | | | Models 6: Average cost per RYO cigarette | | | |
| --- | --- | --- | --- | --- | --- | --- | --- | --- | --- | --- | --- | --- |
|  | B | 95%CI | | P | B | 95%CI |  | P | B | 95%CI |  | p |
|  |  | Lower | Upper |  |  | Lower | Upper |  |  | Lower | Upper |  |
| Step level change 1 for FM (March 2010 – December 2012) | -5.2 | -13.6 | 3.9 | 0.255 | 0.4 | -4.1 | 5.0 | 0.880 | 6.4 | <0.1 | 13.1 | 0.050 |
| Step level change 2 for FM (March 2012 – December 2019) | -5.4 | -13.7 | 3.7 | 0.235 | 4.8 | -0.1 | 9.9 | 0.056 | 0.5 | -5.0 | 6.3 | 0.856 |
| Step level change 1 RYO (March 2011-December 2019) | -9.6 | -17.6 | -0.7 | 0.034 | 5.8 | 1.0 | 10.9 | 0.018 | 3.4 | -3.6 | 10.9 | 0.354 |
| Step level change 2 RYO (March 2016-December 2019) | -6.4 | -14.8 | 2.9 | 0.174 | 5.8 | 0.4 | 11.5 | 0.034 | 7.8 | 2.8 | 13.0 | 0.002 |
| Step level change 3 RYO (November 2017-December 2019) | -4.2 | -12.7 | 5.2 | 0.369 | 8.4 | 3.4 | 13.6 | 0.001 | 10.0 | 2.9 | 17.4 | 0.005 |
| Step level change 4 RYO (November 2018-December 2019) | -10.5 | -18.4 | -2.0 | 0.017 | -5.2 | -9.4 | -0.7 | 0.023 | 3.1 | -3.5 | 10.3 | 0.365 |
| Model specification  AR  MA  SAR  SMA | ARIMAX(0,1,1)_12_  -0.844 | -0.926 | -0.761 | <0.001 | ARIMAX(0,1,1)_12_  -1.091 -1.171 -0.994 <0.001 | | | | ARIMAX(0,1,1)_12_  -1.000 -1.038 -0.960 <0.001 | | | |

Supplementary Table 11: Sensitivity analysis – association between tax increases and overall average cigarette consumption stratified by exclusive FM and RYO cigarettes (fully adjusted)

|  | Model 1: Average FM cigarette consumption | | | | Models 2: Average RYO cigarette consumption | | | |
| --- | --- | --- | --- | --- | --- | --- | --- | --- |
|  | B | 95%CI | | p | B | 95%CI | | P |
|  |  | Lower | Upper |  |  | Lower | Upper |  |
| Step level change 1 for FM (March 2010 – December 2012) | 1.7 | -11.6 | 17.0 | 0.810 | -10.1 | -13.7 | -22.5 | 0.155 |
| Step level change 2 for FM (March 2012 – December 2019) | -1.4 | -12.6 | 12.4 | 0.829 | -9.6 | -15.1 | -22.6 | 0.198 |
| Pulse effect for RYO (March 2011) | -20.6 | -37.9 | 1.6 | 0.067 | -19.2 | -37.9 | -35.9 | 0.071 |
| Pulse effect for RYO (March 2016) | -2.7 | -23.9 | 24.6 | 0.831 | -29.4 | -23.9 | 2.6 | 0.029 |
| Pulse effect for RYO (November 2017) | -1.1 | -22.8 | 26.6 | 0.927 | -4.5 | -22.8 | -17.2 | 0.712 |
| Pulse effect for RYO (November 2018) | -7.0 | -27.4 | 19.2 | 0.568 | -22.8 | -27.4 | -2.8 | 0.085 |
| Model specification  AR  MA  SAR  SMA | ARIMAX(0,1,1)_12_  -1.155 | -1.287 | -1.024 | <0.001 | ARIMAX(0,1,1)  -1.245 | -1.378 | -1.115 | <0.001 |

Supplementary Table 12: Association between tax increases and overall smoking prevalence and prevalence of quit success using quarterly data (fully adjusted)

|  | Model 1: Smoking prevalence | | | | Model 2: Quit success prevalence | | | | Model 3: Average cigarette consumption | | | | Model 4: Average cost per RYO cigarette | | | |
| --- | --- | --- | --- | --- | --- | --- | --- | --- | --- | --- | --- | --- | --- | --- | --- | --- |
|  | B | 95%CI | | P | B | 95%CI |  | p | B | 95%CI |  | P | B | 95%CI |  | p |
|  |  | Lower | Upper |  |  | Lower | Upper |  |  | Lower | Upper |  |  | Lower | Upper |  |
| Step level change 1 for FM (March 2010 – December 2012) | -2.6 | -11.4 | 7.2 | 0.591 | -3.6 | -16.8 | 11.8 | 0.061 | -4.9 | -15.9 | 7.4 | 0.420 | 4.7 | -4.4 | 14.6 | 0.323 |
| Step level change 2 for FM (March 2012 – December 2019) | -5.7 | -14.3 | 3.8 | 0.232 | 29.5 | 14.3 | 46.7 | <0.001 | -7.7 | -18.4 | 4.4 | 0.204 | 0.8 | -8.3 | 10.8 | 0.867 |
| Pulse effect for RYO (March 2011) | -18.9 | -35.0 | 1.3 | 0.064 | -48.8 | -79.5 | 27.9 | 0.152 | -15.8 | -35.9 | 10.6 | 0.216 | 18.2 | -15.5 | 65.4 | 0.328 |
| Pulse effect for RYO (March 2016) | -3.1 | -22.7 | 21.4 | 0.783 | 54.3 | -35.4 | 269.0 | 0.330 | 5.2 | -20.4 | 39.0 | 0.722 | 3.5 | -25.8 | 44.4 | 0.839 |
| Pulse effect for RYO (November 2017) | 7.0 | -14.8 | 34.5 | 0.560 | -47.7 | -78.1 | 24.6 | 0.143 | -0.1 | -24.6 | 32.2 | 0.993 | -3.7 | -31.3 | 34.9 | 0.825 |
| Pulse effect for RYO (November 2018) | 2.5 | -18.3 | 28.5 | 0.202 | -42.4 | -75.9 | 37.4 | 0.213 | 0.9 | -23.4 | 33.2 | 0.945 | -8.3 | -34.9 | 29.1 | 0.618 |
| Model specification  AR  MA  SAR  SMA | ARIMAX(0,1,1)_12_  -0.416 | -0.703 | -0.129 | 0.004 | ARIMAX(0,1,1)_12_  -1.000 -1.115 -0.887 <0.001 | | | | ARIMAX(0,1,1)_12_  -0.375 | -0.642 | -0.106 | 0.006 | ARIMAX(0,1,1)_12_  -0.728 | -0.896 | -0.560 | <0.001 |

**Methods**

***Participants***

Data were aggregated on 274,890 participants; of whom 19.6% (95%CI 19.5 to 19.8) were smokers and 21.6% (95%CI 21.4 to 21.7) were past year smokers. Of the past year smokers, 35.5% (95%CI 35.1 to 35.9) had made a quit attempt in the past year, 15.8% (95%CI 15.2 to 16.3) of which were successful.

***Study design***

Data come from the Smoking Toolkit Study (STS), a monthly survey of a representative sample of the population in England aged 16+ (Jennifer A Fidler et al., 2011). It has been collecting data on smoking patterns among smokers and recent ex-smokers since November 2006. The STS involves monthly household surveys using a random location sampling design, with initial random selection of grouped output areas (containing ~300 households), stratified by sociodemographic characteristic and region. Interviewers then select houses within these areas that are most likely to fulfil quotas and conduct face-to-face computer-assisted interviews with one member per household. The quotas are tailored to the output area and the probability of certain groups being at home. Participants from the STS appear to be representative of the population in England, having similar socio-demographic composition as other large national surveys, such as the Health Survey for England.

***Data on outcome variables***

*Prevalence of smoking, quit attempts and the success of quit attempts*

Smoking prevalence, quit attempt prevalence and the prevalence of the success of those quit attempts were derived using the following questions:

1. “Which of the following best applies to you? a) I smoke cigarettes (including RYO) every day; b) I smoke cigarettes (including RYO), but not every day; c) I do not smoke cigarettes at all, but I do smoke tobacco of some kind (e.g. pipe or cigar); d) I have stopped smoking completely in the last year; e) I stopped smoking completely more than a year ago; f) I have never been a smoker (i.e. smoked for a year or more)”.
2. [Past-year smokers only] “How many serious attempts to stop smoking have you made in the last 12 months? By serious attempt I mean you decided that you would try to make sure you never smoked again. Please include any attempt that you are currently making and please include any successful attempt made within the last year”.
3. [Past-year smoker who have made a quit attempt only] “How long did your most recent serious quit attempt last before you went back to smoking?”

The prevalence of current cigarette smoking was calculated as the proportion of respondents who report ia) or ib). The prevalence of quit attempts in each month was calculated as the number of respondents who reported having made one or more quit attempts in the past 12 months divided by the number of past year smokers in response to ii).

The quit success rate in each month was calculated as the number of respondents reporting that they were still not smoking divided by the number reporting having made a quit attempt in response to iii).

Two sensitivity analyses used the a) smoking prevalence of predominant FM cigarette and b) smoking prevalence of predominant RYO cigarette instead of overall cigarette smoking prevalence. Cigarette smokers were asked how many RYO cigarettes they smoked per day. The prevalence of predominant roll-your-own smoking in each month was calculated as the proportion of participants who report that ≥50% of the cigarettes they smoked are roll-your-own cigarettes (Jackson, Shahab, Garnett, & Brown, 2020). The prevalence of predominant FM cigarette smoking in each month was calculated as the proportion of participants who report that <50% of the cigarettes they smoked were roll-your-own cigarettes.

*Per capita cigarette consumption*

Smokers were asked on average how many cigarettes they smoked per day. The per capita consumption per day was then calculated as the summation of cigarettes smoked per day (which was set to zero for all non-current smokers i.e. non-smokers and ex-smokers) divided by the entire population. Consumption within the population rather than among smokers was used to reflect quitting, i.e. a reduction may reflect an increase in recent ex-smokers without a change in consumption by continuing smokers.

The mean per capita consumption was calculated separately per day for i) FM cigarettes and ii) RYO cigarettes. FM was calculated as the summation of cigarettes smoked per day among those exclusively smoking FM cigarettes divided by cigarette consumption for the entire population, which was set to 0 for all non-current exclusive FM cigarette smokers; RYO as the summation of cigarettes smoked per day among those exclusively smoking RYO cigarettes divided by cigarette consumption for the entire population, which was set to 0 for all non-current exclusive RYO cigarette smokers.

*Cost per cigarette (£)*

To assess self-reported cost per cigarette, current smokers were asked: ‘On average about how much per week do you think you spend on cigarettes or tobacco?’ and the number of cigarettes they smoked per week was calculated (including hand‐rolled). Smokers’ average expenditure of smoking (in £/week) was derived from the following liberal assumptions for upper and lower estimates of plausible levels of consumption and expenditure per week (Kuipers et al., 2019): (1) consumption of a maximum of 560 cigarettes per week; (2) spending does not exceed £280 per week; and (3) single cigarette expenditure between £0.05 and £1. The cost of smoking was adjusted for inflation using Consumer Prices Index data of all items from the Office for National Statistics, with January 2007 as the baseline/reference. The total cost per week was divided by the total number of cigarettes smoked per week to give as estimate of cost per cigarette. Cost per cigarette (£) was derived separately for those exclusively smoking FM cigarettes and those exclusively smoking RYO cigarettes. Cost per cigarette was stratified in this way since any associations between tax increases and cigarette cost may be diluted by smokers switching between FM and RYO cigarettes, depending on the nature of the tax increase.

***Data on covariates***

Covariates were chosen based on there being a plausible relationship between the variables and the outcomes of interest. The covariates included several tobacco control policies combined into a composite score (coded 1 during the month they were implemented and 0 in other months). These were the introduction of a smoking ban in July 2007, change in the minimum age of sale of cigarettes in October 2007, pictorial health warnings on product packaging introduced in October 2008, partial (i.e. supermarket) tobacco point-of-sale display ban introduced in England in April 2012, the full point-of-sale ban in April 2015 and the tobacco products directive/plain packaging in May 2016. There is substantial evidence for the association between these population-level tobacco control policies and quitting activities. The introduction of a smoking ban in July 2007 was associated with a significant temporary increase in the percentage of smokers attempting to stop (Hackshaw, McEwen, West, & Bauld, 2010) and the change in the minimum age of sale of cigarettes in October 2007 resulted in a greater fall in prevalence in 16-17 year olds (J. A. Fidler & West, 2010). Pictorial health warnings on product packaging have been shown to promote smoking cessation(Hammond, 2011). An evaluation of the partial tobacco point-of-sale display ban introduced in England in April 2012 (only for large retail shops, with smaller shops requiring implementation by April 2015) found evidence for a decline in smoking prevalence (Kuipers et al., 2017).

We also included monthly tobacco mass media expenditure (in million £) which was obtained from Public Health England. Total spending on campaigns was calculated for each month and included spending on ‘Smokefree’ campaigns, Stoptober campaigns and Health Harms campaigns. Spend included TV, radio, print, cinema and on‐line advertisements. In England, tobacco control mass media campaigns have been run as part of a national tobacco control programme. Spending was almost completely suspended in 2010 and then reintroduced in 2011 at a much lower level. Time series analyses have shown that such cuts on tobacco mass media expenditure are associated with a reduction in use of smoking cessation support (Langley et al., 2014) and that higher monthly expenditure on tobacco control mass media campaigns in England is associated with a higher rate of quit success (Beard, Jackson, West, Kuipers, & Brown, 2020).

***Data on explanatory variables***

For the overall primary analysis we modelled two step level changes for the tax increases on FM cigarettes [See data file https://osf.io/kz3bc/ columns R and S]. For both, the first segment of data covers a period of only inflation adjustment with no real increases in tobacco tax (January 2007 until February 2010), this period was thus coded 0. The first step level change was for the period of above inflation tax increases between March 2010 until December 2019. This period was coded as 1. The second step level change reflected the larger tax increase of 5% in March 2012 and was coded 0 before February 2012 and 1 thereafter. We also included four pulse effects for the tax increases on RYO cigarettes (coded 1 during the month they were first implemented and 0 in other months) [See data file https://osf.io/kz3bc/ columns T, U, V and W]. The decision was made a priori to measure these as pulse effects as they were not followed by further immediate tax increases

As a sensitivity analysis, we also modelled the 5% increase in tax on FM cigarettes as a pulse effect which was coded 1 for March 2012 and 0 for all other months [See data file https://osf.io/kz3bc/ column X]. We also included a step level effect for the four time points in which increases occurred for RYO tobacco (coded 0 before the tax change and 1 after) [See data file https://osf.io/kz3bc/ columns Y, Z, AA, AB]. Sensitivity analyses stratified results according to the use of FM cigarettes and RYO cigarettes.

In further sensitivity analysis, we also stratified the models by SES. SES was measured using the Social-Grade Classification Tool, which categorises individuals into one of five social grades: AB, C1, C2, D and E. Grades AB and C1 were classified as ‘non-manual’ or higher SES and Grades C2 to E were classified as ‘manual’ or lower SES occupational groups.

We modelled the date of the announcement of the tax increases rather than the implementation. This is because tobacco industry tactics are implemented around the announcement date: increasing prices on top of tax increases, so that both the price and tax increase are passed on to consumers (known as overshifting), absorbing the tax increase so it is not passed on to consumers (undershifting) or passing the tax increase on to consumers in full (fully shifting) (Partos et al., 2020). Any impact of these on smoking behaviour would therefore be detected.

Temporary pulse effects may more reflect the announcement forewarning smokers about tax changes, while step level changes may reflect more of the impact of tax increases themselves or tobacco industry tactics.

**Analysis**

***Amendments***

In the original analysis plan we had included ever smoking (as an indicator of uptake) as an outcome variable. However, effect size estimates from the models including ever smoking appeared implausibly large. After internal review by colleagues who are not authors, we decided that ever smoking among the entire population was not an appropriate measure of uptake as most smoking is established by young adulthood. We considered including ever smoking among those aged 16 to 24 but the data were too variable over time with large confidence intervals due to the smaller sample size, and therefore models would have been imprecise. This data is provided in the updated data frame on the Open Science Framework along with the results of the pre-planned analysis for ever smoking (<https://osf.io/kz3bc/>).

During the same internal review it was also decided to add new outcome variables in the primary and sensitivity analyses: i) average population cigarette consumption (primary analysis) i) average population FM cigarette consumption per day (sensitivity analysis), ii) average population RYO cigarette consumption per day (sensitivity analysis), iii) average price per FM cigarette (primary analysis) and iv) average price per RYO cigarette (primary analysis). These variables may be more sensitive to FM/RYO-specific tax increases and have been used widely in previous literature (Callison & Kaestner, 2014; Chaloupka, Yurekli, & Fong, 2012; DeCicca & McLeod, 2008; Kuipers et al., 2019).

Following concern that the size of the significant pulse and step level effects may have been sensitive to randomness in the monthly data, it was also decided to run additional unplanned sensitivity analyses using smoothed data at the quarterly rather than monthly level.

***Primary analysis***

All data were analysed in R studio. The data frame and analysis plan were pre-registered on the Open Science Framework (https://osf.io/kz3bc/).

We used an interrupted time series analysis to account for autocorrelation among monthly observations by fitting Autoregressive Integrated Moving Average with Exogeneous Input (ARIMAX) models (Chan, Ripley, Chan, & Chan, 2020) . ARIMAX is an extension of autoregressive integrated moving average analysis (ARIMA), which produces forecasts based upon prior values in the time series analysis (AR terms) and the errors made by previous predictions (MA terms). Both adjusted and unadjusted models are reported in this paper.

For the primary analysis we modelled step level changes as a result of the rise in tax on FM cigarettes and pulse effects as a result of the rise in tax on RYO in two ways. We prespecified modelling the tax increases in this way as: 1) tax increases for RYO were infrequent and therefore we hypothesised temporary rather than longer term changes and 2) tax increases for FM were frequent and consistent over time and therefore were better modelled as step changes. In the analysis these models were also the most stable and provided an adequate fit.

We first modelled the announcement of the tax riseas abrupt sustained step level changes (i.e. an immediate decline in prevalence which is maintained over the rest of the series) and abrupt temporary pulse effects (i.e. an immediate decline in prevalence which is maintained for one month before returning immediately to previous levels). This was done by including zero-order transfer functions in the model i.e. modelling them as dummy covariates. We also modelled them as a gradual sustained step level changes (i.e. a gradual decline in prevalence which is then maintained over the rest of the series) and temporary pulse effects which decay gradually over time (i.e. an immediate decline in prevalence which after 1 month returns gradually to previous levels). This was done by including them with first-order transfer functions in the model i.e. modelling them as covariates that affect the time series in terms of an ARMA filter of order (1,0). These models were compared using the Akaike Information Criterion (AIC). A temporary pulse effect which decayed over time (i.e. an impact which defused slowly throughout the study period or in other words a first-order transfer function) provided the best model fit for the four rises in tax on RYO cigarettes when predicting overall quit success prevalence and average cost per FM cigarettes (see Supplementary Table 1). Results when modelling abrupt temporary pulse effects for the four rises in tax on RYO cigarettes when predicting overall quit success prevalence and average cost per FM cigarette, are given in Supplementary Table 2.

As a sensitivity analysis, we modelled the 5% increase in tax on FM cigarettes as a pulse effect and step level effects for the four time points in which increases occurred for RYO tobacco. Modelling the 5% increase in tax on FM cigarettes did not significantly improve model fit, as assessed by the AIC. These results are therefore given in Supplementary Table 9 but are not reported in the results. Results were similar to the primary analysis. Modelling the pulse effects for the rise in tax on RYO cigarettes instead as step level changes did significantly improve model fit for all outcomes expect for the impact on per capita cigarette consumption and prevalence of quit attempts. However, confidence intervals around the effect sizes were large and the effect sizes elevated (with some sign flipping from the primary analysis). This likely reflects poor model stability due to sample size and over parameterization. As such, these results are also only presented in Supplementary material (see Supplementary Table 10).

Standard recommended procedures were used to select the ARIMAX models (Beard et al., 2019). First, we assessed each time series for outlying values (using box plots) which may bias the results. Secondly, plots of the differenced data and unit root tests (i.e. Osborn-Chui-Smith-Birchenhall test (OCBS) and Kwiatkowski, Phillips, Schmidt, and Shin (KPSS) test) were used to determine the number of seasonal and non-seasonal differences required for the time series analyses to be made stationary.

Next, in order to determine the initial values of the AR and MA terms for the baseline models, the autocorrelation function (ACF) and partial autocorrelation function (PACF) were assessed. These suggested the presence of some lower order autocorrelation (either MA(1) or AR(1)). . Additional models with various fitted AR and MA terms were then compared to this baseline model using the AIC up to an order of 3.The model with lower AIC values was selected. Next the ACF for the residuals of the best fitting model and Ljung-Box test for white noise were used to statistically evaluate the degree to which the residuals were free of serial correlation (thus the need for additional MA/AR seasonal or non-seasonal terms). The final model residuals were also assessed for normality and that the coefficients of the correlation terms were significant and fell within the bounds of stationarity and invertibility.

There was no evidence of additional residual autocorrelation and therefore the need for higher order non-seasonal AR or MA terms. There was also no evidence across the models for additional residual seasonal autocorrelation.

To identify the most appropriate transfer function for the continuous explanatory variables, we checked the sample cross correlation function and compared models with varying lags using the Akaike information criterion.

A log transformation was also applied to the output series and tobacco control mass media expenditure, thus coefficients were transformed so that a 1-unit change in the categorical variables (step level changes and pulse effects) corresponded to an expected change in the outcome variable of 100*(exp(B)-1) %.

Bayes factors were calculated for non-significant findings for the primary analysis in R using code described by Dienes (Beard, Dienes, Muirhead, & West, 2016; Dienes, 2014). This helps to determine if there is evidence for the null hypothesis of no difference or the data are insensitive to detect an effect. The expected effect size corresponded to a percentage point decline of 0·745 (or increase in the case of quit attempts and quit success) identified in a previous study. This absolute reduction in smoking prevalence was equivalent to a relative reduction in overall smoking prevalence of 4·2%. A half-Gaussian distribution was specified. The same predicted reduction was also used for per capita cigarette consumption per day and the average cost per cigarette, while a relative increase in quit attempts and quit success of 4.2% was assumed. Bayes factor robustness regions are also reported.

***Missing data***

No data were collected in December 2008 from the Smoking Toolkit study. Variables during this period were calculated as an average of the month before and the month after**.** Current smokers were only asked the percentage of cigarettes they smoked which were RYO from November 2007. Sensitivity analyses comparing prevalence of predominant FM cigarette smokers and prevalence of predominant RYO cigarette smokers were therefore run from November 2007 until December 2019. Questions on the cost of cigarettes were only collected between October 2007 and June 2009 and then from August 2010 onwards. Missing values for these periods were imputed using Kalman Smoothing for univariate time series data.

***Assumptions and outliers***

One outlier was identified for the series of average price of exclusive RYO cigarettes (April 2008) using (1) the ‘tsoutliers’ package (López-de-Lacalle, 2019), which implements an iterative procedure of anomaly identification and model estimation based on the approach described in Chen & Liu [51] and (2) box-plots. The suggested replacement value from the tsoutliers package did not differ substantially (i.e. £0.19 versus replacement £0.12) and was therefore deemed to be a true value and retained in the primary analysis. Replacement of the outlier did not change the model results or assumptions.

There was no evidence that the continuous covariate, mass media spend, violated the assumption of weak exogeneity (i.e., Y can depend on the lagged values of X but the reverse must not be true) between the input and the output series. This was assessed using the Granger causality test. The coefficients of the correlation terms in the final model were all significantly and largely fell within the bounds of stationarity and invertibility, which ensures the series is stationary around its mean. The Ljung-Box test for white noise showed the residuals of the final models were free of serial correlation and the residuals followed a normal distribution.

**References**

Beard, E., Dienes, Z., Muirhead, C., & West, R. (2016). Using Bayes factors for testing hypotheses about intervention effectiveness in addictions research. *Addiction, 111*(12), 2230-2247.

Beard, E., Jackson, S. E., West, R., Kuipers, M. A. G., & Brown, J. (2020). Population-level predictors of changes in success rates of smoking quit attempts in England: a time series analysis. *Addiction, 115*(2), 315-325. doi:<https://doi.org/10.1111/add.14837>

Beard, E., Marsden, J., Brown, J., Tombor, I., Stapleton, J., Michie, S., & West, R. (2019). Understanding and using time series analyses in addiction research. *Addiction, 114*(10), 1866-1884.

Callison, K., & Kaestner, R. (2014). Do higher tobacco taxes reduce adult smoking? New evidence of the effect of recent cigarette tax increases on adult smoking. *Economic Inquiry, 52*(1), 155-172.

Chaloupka, F. J., Yurekli, A., & Fong, G. T. (2012). Tobacco taxes as a tobacco control strategy. *Tobacco control, 21*(2), 172-180.

Chan, K.-S., Ripley, B., Chan, M. K.-S., & Chan, S. (2020). Package ‘TSA’. *R package version, 1*.

DeCicca, P., & McLeod, L. (2008). Cigarette taxes and older adult smoking: Evidence from recent large tax increases. *Journal of health economics, 27*(4), 918-929.

Dienes, Z. (2014). Using Bayes to get the most out of non-significant results. *Frontiers in psychology, 5*, 781.

Fidler, J. A., Shahab, L., West, O., Jarvis, M. J., McEwen, A., Stapleton, J. A., . . . West, R. (2011). 'The smoking toolkit study': a national study of smoking and smoking cessation in England. *BMC Public Health, 11*(1), 1-9.

Fidler, J. A., & West, R. (2010). Changes in smoking prevalence in 16-17-year-old versus older adults following a rise in legal age of sale: findings from an English population study. *Addiction, 105*(11), 1984-1988. doi:10.1111/j.1360-0443.2010.03039.x

Hackshaw, L., McEwen, A., West, R., & Bauld, L. (2010). Quit attempts in response to smoke-free legislation in England. *Tobacco control, 19*(2), 160-164.

Hammond, D. (2011). Health warning messages on tobacco products: a review. *Tobacco control, 20*(5), 327-337.

Jackson, S. E., Shahab, L., Garnett, C., & Brown, J. (2020). Trends in and correlates of use of roll-your-own cigarettes: a population study in England 2008–2017. *Nicotine and Tobacco Research, 22*(6), 942-949.

Kuipers, M. A., Beard, E., Hitchman, S. C., Brown, J., Stronks, K., Kunst, A. E., . . . West, R. (2017). Impact on smoking of England's 2012 partial tobacco point of sale display ban: a repeated cross-sectional national study. *Tobacco control, 26*(2), 141-148.

Kuipers, M. A., Partos, T., McNeill, A., Beard, E., Gilmore, A. B., West, R., & Brown, J. (2019). Smokers’ strategies across social grades to minimise the cost of smoking in a period with annual tax increases: evidence from a national survey in England. *BMJ open, 9*(6), e026320.

Langley, T., Szatkowski, L., Lewis, S., McNeill, A., Gilmore, A. B., Salway, R., & Sims, M. (2014). The freeze on mass media campaigns in E ngland: a natural experiment of the impact of tobacco control campaigns on quitting behaviour. *Addiction, 109*(6), 995-1002.

López-de-Lacalle, J. (2019). R Package tsoutliers.

Partos, T. R., Hiscock, R., Gilmore, A. B., Branston, J. R., Hitchman, S., & McNeill, A. (2020). Impact of tobacco tax increases and industry pricing on smoking behaviours and inequalities: a mixed-methods study.
